# Integration analysis of PacBio SMRT- and Illumina RNA-seq reveals candidate genes and pathway involved in selenium metabolism in hyperaccumulator *Cardamine violifolia*

**DOI:** 10.1186/s12870-020-02694-9

**Published:** 2020-10-27

**Authors:** Shen Rao, Tian Yu, Xin Cong, Feng Xu, Xiaozhuo Lai, Weiwei Zhang, Yongling Liao, Shuiyuan Cheng

**Affiliations:** 1 College of Horticulture and Gardening, Yangtze University, Jingzhou, 434025 China; 2 National R&D for Se-rich Agricultural Products Processing Technology, Wuhan Polytechnic University, Wuhan, 430023 China; 3 Enshi Se-Run Health Tech Development Co., Ltd, Enshi, 445000 China; 4 National Selenium Rich Product Quality Supervision and Inspection Center, Enshi, 445000 Hubei China

**Keywords:** *Cardamine violifolia*, Full-length transcriptome, lncRNA, RNA-seq, Selenium metabolism, Selenate treatment

## Abstract

**Background:**

*Cardamine violifolia*, native to China, is one of the selenium (Se) hyperaccumulators. The mechanism of Se metabolism and tolerance remains unclear, and only limited genetic information is currently available. Therefore, we combined a PacBio single-molecule real-time (SMRT) transcriptome library and the Illumina RNA-seq data of sodium selenate (Na_2_SeO_4_)-treated *C. violifolia* to further reveal the molecular mechanism of Se metabolism.

**Results:**

The concentrations of the total, inorganic, and organic Se in *C. violifolia* seedlings significantly increased as the Na_2_SeO_4_ treatment concentration increased. From SMRT full-length transcriptome of *C. violifolia*, we obtained 26,745 annotated nonredundant transcripts, 14,269 simple sequence repeats, 283 alternative splices, and 3407 transcription factors. Fifty-one genes from 134 transcripts were identified to be involved in Se metabolism, including transporter, assimilatory enzyme, and several specific genes. Analysis of Illumina RNA-Seq data showed that a total of 948 differentially expressed genes (DEGs) were filtered from the four groups with Na_2_SeO_4_ treatment, among which 11 DEGs were related to Se metabolism. The enrichment analysis of KEGG pathways of all the DEGs showed that they were significantly enriched in five pathways, such as hormone signal transduction and plant-pathogen interaction pathways. Four genes related to Se metabolism, *adenosine triphosphate sulfurase 1*, *adenosine 5′-phosphosulfate reductase 3*, *cysteine (Cys) desulfurase 1*, and *serine acetyltransferase 2*, were regulated by lncRNAs. Twenty potential hub genes (e.g., *sulfate transporter 1;1*, Cys synthase, *methionine gamma-lyase,* and *Se-binding protein 1*) were screened and identified to play important roles in Se accumulation and tolerance in *C. violifolia* as concluded by weighted gene correlation network analysis. Based on combinative analysis of expression profiling and annotation of genes as well as Se speciation and concentration in *C. violifolia* under the treatments with different Na_2_SeO_4_ concentrations, a putative Se metabolism and assimilation pathway in *C. violifolia* was proposed.

**Conclusion:**

Our data provide abundant information on putative gene transcriptions and pathway involved in Se metabolism of *C. violifolia*. The findings present a genetic resource and provide novel insights into the mechanism of Se hyperaccumulation in *C. violifolia*.

**Supplementary information:**

**Supplementary information** accompanies this paper at 10.1186/s12870-020-02694-9.

## Background

*Cardamine violifolia*, a species belonging to the Brassicaceae family, is mainly distributed in Enshi and Huping Mountains of China, and has been reported to be one of the selenium (Se) hyperaccumulating plants [1]. To date, three different Se-accumulating *Cardamine* species from China have been investigated. The main forms of accumulated Se were organic seleno-compounds, but the speciation of Se differed from one to another: selenocystine (SeCys2) for *C. hupingshanensis* [2], selenocysteine (SeCys) and methyl SeCys (MeSeCys) for *C. enshiensis* [3] and selenolanthionine for *C. violifolia* [1], respectively. An ensuing study demonstrated the remarkable differences in tolerance, accumulation, location, and speciation of Se between *C. violifolia* and its non-accumulating sister species *C. pratensis* [4]. These studies mainly have so far focused on the forms, translocation, distribution of Se in different tissues of *Cardamine* species, yet few studies have addressed the molecular mechanism of Se metabolism and tolerance in *C. violifolia*. Recently, transcriptome analysis by Zhou et al. (2018) suggested that selenate (SeO_4_^2−^) is the initial Se compound metabolized by *C. hupingshanensis*, and that Se toxification can be alleviated via the transamination and degradation of several Se-containing compounds [5]. However, molecular information on metabolism and tolerance of Se in *C. violifolia* remains scarce.

The most common forms of Se found in nature are selenite and selenate [6]. In anaerobic environments, selenite is the main form of Se, and it is absorbed by plants via phosphate transporters [7, 8]. Unfortunately, this phenomenon has not been explained well thus far. Selenate is the dominant Se form in oxic soils, which include most cultivated soils [8]. Unlike selenite, selenate is taken up via sulfate transporters (Sultr) in plants [6]. The uptake and metabolic processed of selenate have been more clearly demonstrated than those of selenite using *Arabidopsis* and the Se hyperaccumulator *Stanleya pinnata* as model plants [8, 9]. Four groups of Sultrs in plants responsible for Se uptake and transportation, including Sultr1, Sultr2, Sultr3, and Sultr4, are found in plants, and each group contains several isoforms [10–12]. After entering into plants, selenate can be assimilated by a series of enzymes that are essentially employed to metabolize sulfate (e.g., adenosine triphosphate sulfurase [APS], adenosine 5′-phosphosulfate reductase [APR], and selenite reductase [SIR]) [13]. Similar to how sulfate is transformed into amino acid (aa), selenate is assimilated into seleno-aa, including SeCys and selenomethionine (SeMet) [14]. However, SeCys and SeMet can nonspecifically replace Cys and Met in proteins and lead to protein dysfunction, thereby toxifying plants [15]. Se hyperaccumulators, such as *S. pinnata* [16] and *Astragalus bisulcatus* [17], have evolved to become capable of removing Se from their tissues. Methylation of SeCys to MeSeCys and further volatilization to dimethyl-diselenide (DMDSe) is the most effective way to detoxify Se. These reactions are catalyzed by SeCys methyltransferase (SMT) and Cys sulfoxide lyase (CSL) [9]. Similar to SeCys, SeMet also can be transformed into methyl-SeMet (MeSeMet) via the mediation of S-adenosyl-L-Met:Met-S-methyltransferase (MMT) and then into a volatile compound, dimethyl-selenide (DMSe) [8]. Besides volatilization, plants maintain another strategy to eliminate Se, that is, the breakdown of SeCys into elemental Se and alanine via the catalysis of SeCys lyase (SL) [18].

In the case of *C. violifolia*, as a noble Se hyperaccumulator, only limited information on the molecular metabolism of Se hyperaccumulation is available and the genomic information of *C. violifolia* regarding the Se metabolism is lacking. The molecular information on *C. violifolia* would help develop and utilize the plant in phytoremediation as well as in the food industry as Se-rich food source. In recent years, single-molecule real-time (SMRT) sequencing technology has been widely applied to full-length (FL) transcriptome sequencing. The sequence length and information of transcript structures in the FL transcriptome data are superior to those in the second generation Illumina RNA-seq data [19]. Therefore, to clarify the molecular mechanism of Se metabolism, integration analysis of PacBio FL transcriptome and the Illumina RNA-seq in *C. violifolia* under the treatments with different selenate concentrations were carried out. This study identified candidate key genes involved in Se metabolism and established a putative pathway of Se metabolism in *C. violifolia*. The findings of this study will provide novel insights into the molecular process of Se assimilation in *C. violifol*ia, a Se hyperaccumulating plant species.

## Results

### Changes in se concentration and physiological indexes of *C. violifolia* grown under different selenate concentrations

**Fig. 1:**
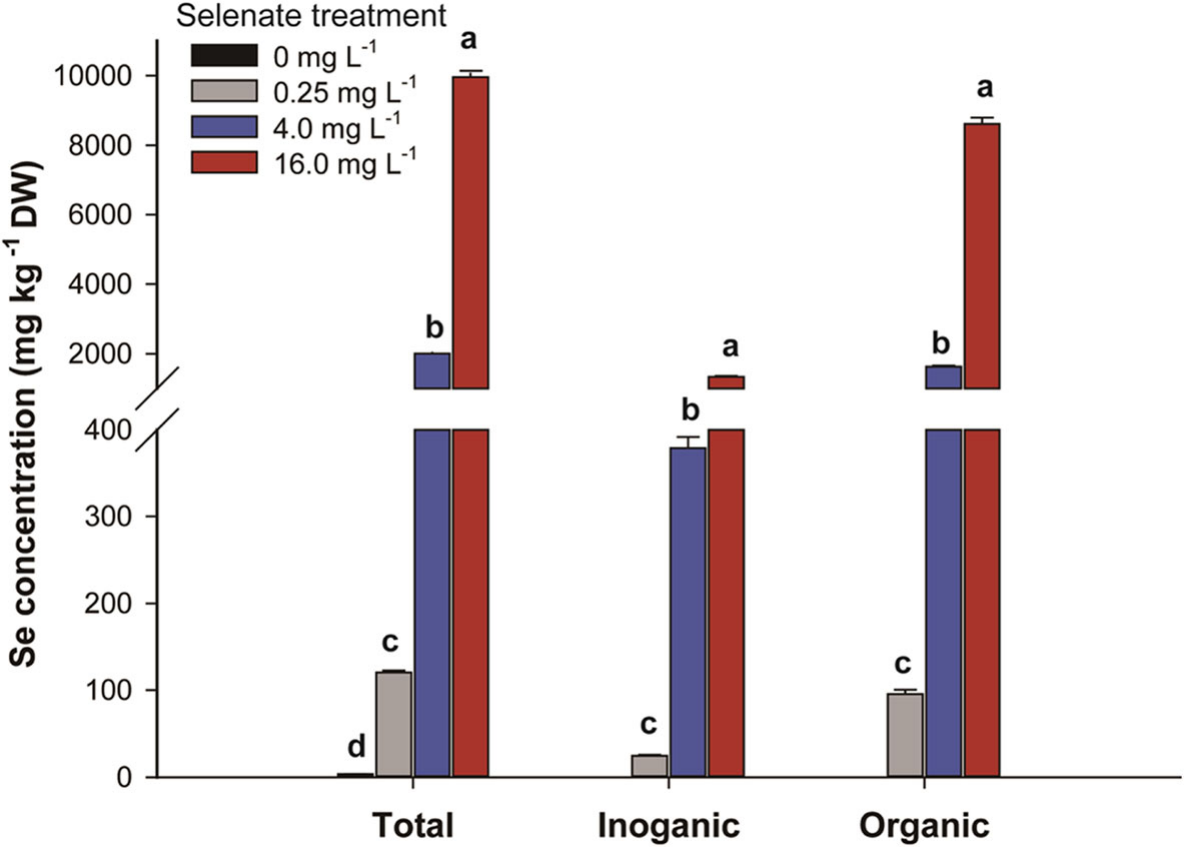
Se concentration from the whole plants of *C. violifolia* treated with different concentrations of Na_2_SeO_4_. Data are shown as mean ± SE (*n* = 3). Means with different letters represent a significant difference at *p* < 0.05

The Se concentration of *C. violifolia* was remarkably affected by sodium selenate (Na_2_SeO_4_) treatment. The concentrations of total, inorganic, and organic Se dramatically increased as the treated Na_2_SeO_4_ concentration was increased (Fig. 1). Noteworthy, the concentrations of total, inorganic, and organic Se in *C. violifolia* peaked to 9955 mg kg^− 1^ DW, 1338 mg kg^− 1^ DW, and 8617 mg kg^− 1^ DW under 16.0 mg L^− 1^ Na_2_SeO_4_ treatment, which was 83-, 54-, and 90-fold of those under 0.25 mg L^− 1^ Na_2_SeO_4_ treatment, respectively. Organic Se made up over 80% of the total Se concentration in *C. violifolia* treated with different concentrations of Na_2_SeO_4_. This result indicates that most of the uptaken selenate in *C. violifolia* is metabolized into organic forms.

The growth of *C. violifolia* was also remarkably influenced by Na_2_SeO_4_ treatments. As shown in Tables 1, 0.25 mg L^− 1^ Na_2_SeO_4_ treatment stimulated the growth of *C. violifolia* as the fresh weight was significantly higher than that of the 0 mg L^− 1^ Na_2_SeO_4_ treatment. There was no significant difference between the 4.0 mg L^− 1^ group and the 0 mg L^− 1^ group on the fresh weight, chlorophyll, free amino acid, and vitamin C content of *C. violifolia*. Whereas for the 16.0 mg L^− 1^ Na_2_SeO_4_ treatment, the fresh weight and chlorophyll content were significantly lower, the free amino acid and vitamin C content were significantly higher, than those in the 0 mg L^− 1^ group. This result indicates that the 0.25 and 4.0 mg L^− 1^ Na_2_SeO_4_ treatment didn’t stress *C. violifolia* plants, while the 16.0 mg L^− 1^ Na_2_SeO_4_ treatment put intense stress on *C. violifolia* plants.

**Table 1:** Effects of different concentrations of Na_2_SeO_4_ on physiological indexes of *C. violifolia*

Na_2_SeO_4_ concentration (mg L^− 1^)	Fresh weight per plant (g)	Chlorophyll content (mg g^− 1^)	Free amino acid content (μg g^− 1^)	Vitamin C content (mg 100 g^− 1^)
0	7.43 ± 0.90 b	0.92 ± 0.13 a	61.91 ± 3.48 b	1.74 ± 0.43 b
0.25	10.30 ± 1.06 a	0.94 ± 0.14 a	63.73 ± 4.72 b	2.52 ± 1.14 ab
4.0	8.01 ± 0.54 ab	1.05 ± 0.04 a	60.22 ± 2.21 b	1.83 ± 0.59 b
16.0	2.76 ± 0.17 c	0.51 ± 0.03 b	92.71 ± 5.65 a	5.04 ± 0.86 a

### Sequencing of *C. violifolia* FL transcriptome

**Fig. 2:**
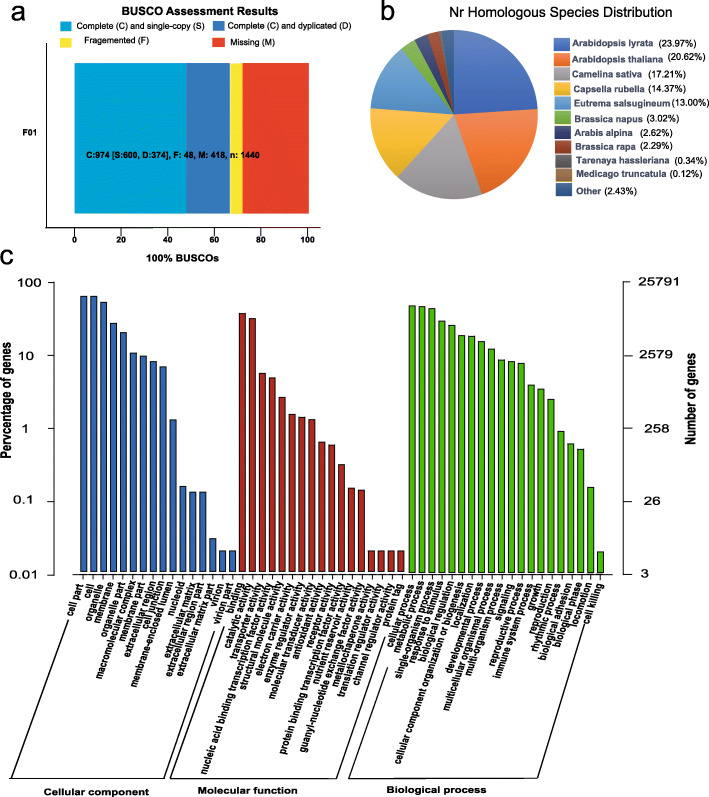
Data integrity assessment and annotation analysis. **a,** BUSCO integrity assessment of the full-length transcriptome data. **b,** Nr homologous species distribution analysis. **c**, GO enrichment statistic of the *C. violifolia* transcriptome

To ensure the quality of the PacBio FL library, we extracted high-quality RNA from the four groups of *C. violifolia* treated with different concentrations of selenate and confirmed that the RNA quality met the requirements of library construction (Additional file 1: Fig. S1). One SMRT cell with a cDNA size of 1–6 k was sequenced on the PacBio RSII platform. A total of 591,350 circular consensus sequences (CCSs) were obtained. Transcript lengths were distributed from 0 to 6000 nucleotides (nt) (Additional file 2: Fig. S2) with an average length of 1807 nt. Among the CCSs obtained, 508,026 (85.9% of the total reads) were FL nonchimeric (FLNC) reads. The FLNC reads were clustered, and 51,182 consensus isoforms with an average length of 1734 nt were obtained, of which 50,900 were high-quality isoforms (Table 2). The low-quality isoforms were then corrected by using the Illumina RNA-seq data to enhance the accuracy of the sequences. Finally, 27,034 nonredundant transcripts were obtained and designated as F01_transcript/xxx. The global expression pattern of the nonredundant transcripts from the 12 samples is shown in Additional file 3: Fig. S3. The integrity of the transcriptome was assessed by BUSCO [20]. In total, 974 of the 1440 expected embryophytic genes were found to be integrative, composed with 600 single-copy and 374 duplicated genes. Among these transcripts, other 418 and 48 genes were missing and fragmented, respectively (Fig. 2a).

**Table 2:** The results of iterative clustering for error correction clustering analysis

Sample	Number of consensus isoforms	Average consensus isoforms read length	Number of polished high-quality isoforms	Number of polished low-quality isoforms	Percent of polished high-quality isoforms (%)
F01	51,182	1734	50,900	277	99.5%

### Coding sequence prediction and transcript annotation

Analysis by TransDecoder software identified a total of 26,707 open reading frames (ORFs), 22,388 of which were complete. The length of the coding proteins of the complete ORFs ranged from 0 to 100 to 1500–1600 aa (Additional file 4: Fig. S4), and 79.1% of the predicted proteins possessed lengths in the range of 100–200, 200–300, 300–400, 400–500, and 500–600 aa. Nonredundant transcripts were searched against several databases including the National Center for Biotechnology Information (NCBI) non-redundant protein sequences (Nr), Kyoto Encyclopedia of Genes and Genomes (KEGG), Protein family (Pfam), Eukaryotic Orthology Groups (KOG), Clusters of Orthologous Groups (COG), the evolutionary genealogy of genes: Nonsupervised Orthologous Groups (egg-NOG), Swiss-Prot, and Gene Ontology (GO). As shown in Table 3, a total of 26,745 transcripts were annotated in the eight databases, of which 26,689 transcripts in Nr, 25,719 transcripts in GO, 12,030 transcripts in COG, 11,864 transcripts in KEGG, 16,982 transcripts in KOG, 23,444 transcripts in Swiss-Prot, and 26,378 transcripts in eggNOG (Additional file 5: Table S1). Analysis of Nr homologous species distributions showed that *C. violifolia* is closely related to other *Brassicaceae* plants, such as *Arabidopsis lyrata* (24.0%), *Arabidopsis thaliana* (20.6%), and *Camelina sativa* (17.2%) (Fig. 2b). In particular, a total of 25,791 transcripts from *C. violifolia* were annotated in 53 GO terms (Fig. 2c). The most abundant terms in the cellular component were cell part (92.4%), cell (92.4%), organelle (75.9%), membrane (37.9%), and organelle part (27.7%). Among molecular functions, the top five terms were binding (52.4%), catalytic activity (44.3%), transporter activity (7.2%), nucleic acid binding transcription factor (TF) activity (6.2%), and structural molecule activity (3.2%). The top five terms among biological processes were cellular process (69.5%), metabolic process (67.8%), single-organism process (63.7%), response to stimulus (42.0.%), and biological regulation (36.6%). The transcripts annotated in the COG database were also classified (Additional file 6: Fig. S5a). A total of 3505 (19.4%), 1779 (9.9%), 1729 (9.6%), 1663 (9.2%), and 1193 (6.6%) transcripts were annotated in general function prediction only, transcription, signal transduction mechanisms, replication, recombination and repair, posttranslational modification, protein turnover, and chaperones, respectively. The most enriched term in the eggNOG functional classification was function unknown, which contained 11,723 transcripts (Additional file 6: Fig. S5b). Other terms, such as post-translational modification, protein turnover, chaperones (8.5%), and signal transduction mechanism (6.8%) were also obtained.

**Table 3:** Statistics of the annotated transcripts

Annotated database	COG	GO	KEGG	KOG	Pfam	Swiss-Prot	eggNOG	Nr	All
Annotation number	12,030	25,791	11,864	16,982	23,444	20,451	26,378	26,689	26,745

### Characterization of the transcripts involved in se metabolism

**Fig. 3:**
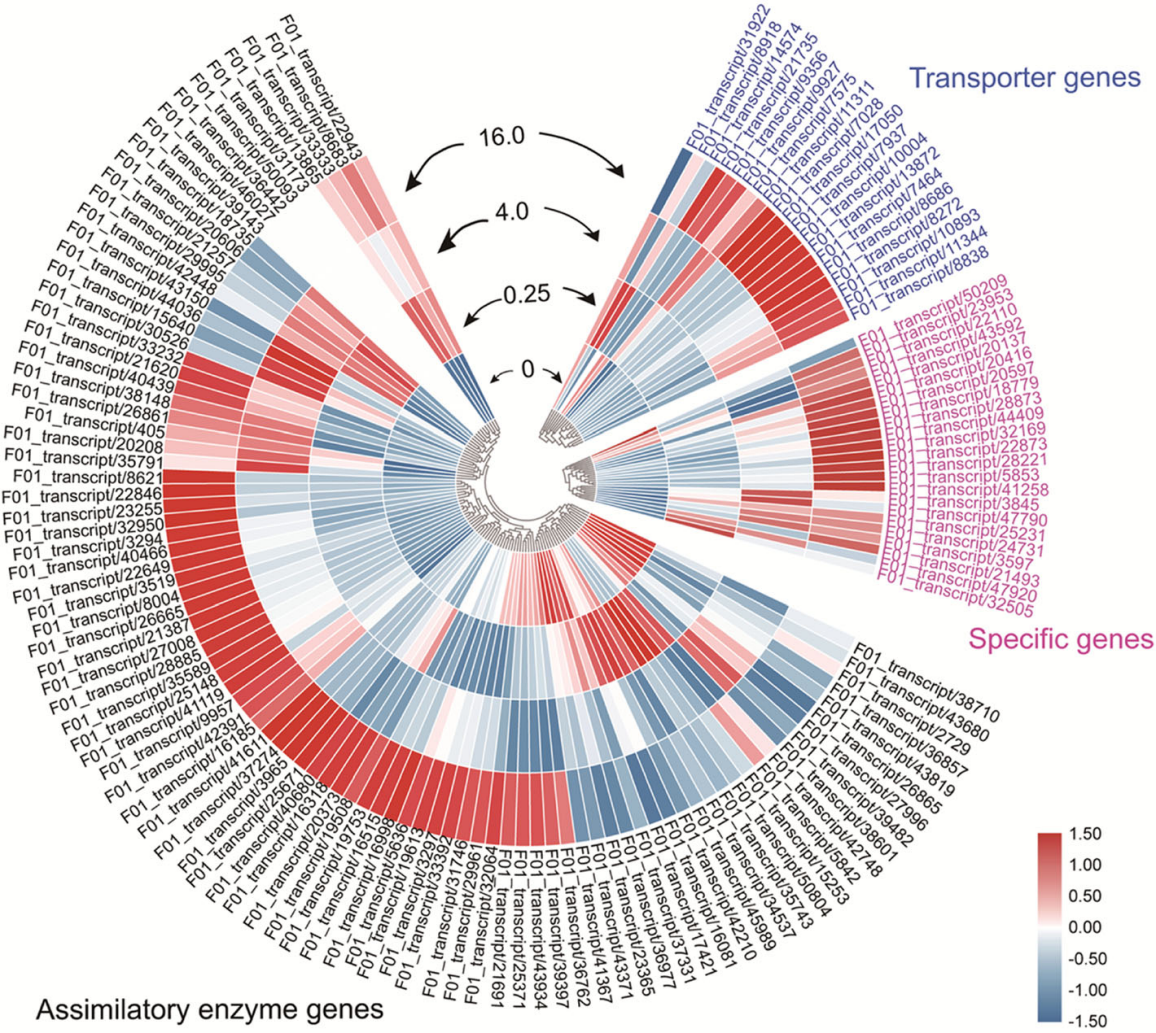
Global expression profile of the screened Se-metabolism related transcripts. The transcripts were categorized into the transporter, assimilatory enzyme, and specific genes with different colors

Fifty-one genes with 134 transcripts involved in Se/Sulfur (S) metabolism were screened from the eight annotation databases (Additional file 7: Table S2). These genes were classified into three categories, namely, transporter, assimilatory enzyme, and specific genes. Eight members of *Sultrs*, including *Sultr1;1*, *Sultr1;2*, *Sultr2;1*, *Sultr2;2*, *Sultr3;2*, *Sultr3;3*, *Sultr3;5*, and *Sultr4;1*, were annotated in Se/S metabolism that are proposed in the uptake and translocation of selenate in *C. violifolia*. The assimilatory enzyme genes contained 31 members such as *APS1–4*, *APR1–5* and *APR7*, *SIR*, *Cys synthase* (*CS*), *Met synthase*, *homocysteine S-methyltransferase* (*HMT*) and *S-adenosyl-L-methionine-dependent methyltransferases* (*MMT*). These genes are involved in S assimilation processes in plants, implying that they may play roles in Se assimilation in *C. violifolia* as well [8, 9]. Besides, 12 genes were identified as specific genes that are related to Se metabolism in *C. violifolia*, which belong to *Cys desulfurase1–2*, *Met-gamma-lyase* (*MGL*), and *Se-binding protein* (*SBP1*) family. These specific genes may play roles in Se metabolism and tolerance in *C. violifolia*. The expression profile analysis of these 134 transcripts from the four treatment groups showed that most of the screened Se-related transcripts enhanced expression in response to Na_2_SeO_4_ treatments, especially in the 16.0 mg L^− 1^ Na_2_SeO_4_ treatment group (Fig. 3). This result further indicates that these Se-related genes may function in Se uptake and metabolism of *C. violifolia* through regulating expression levels.

### Analysis of differentially expressed genes (DEGs) of *C. violifolia* treated with different concentrations of Na_2_SeO_4_

**Fig. 4:**
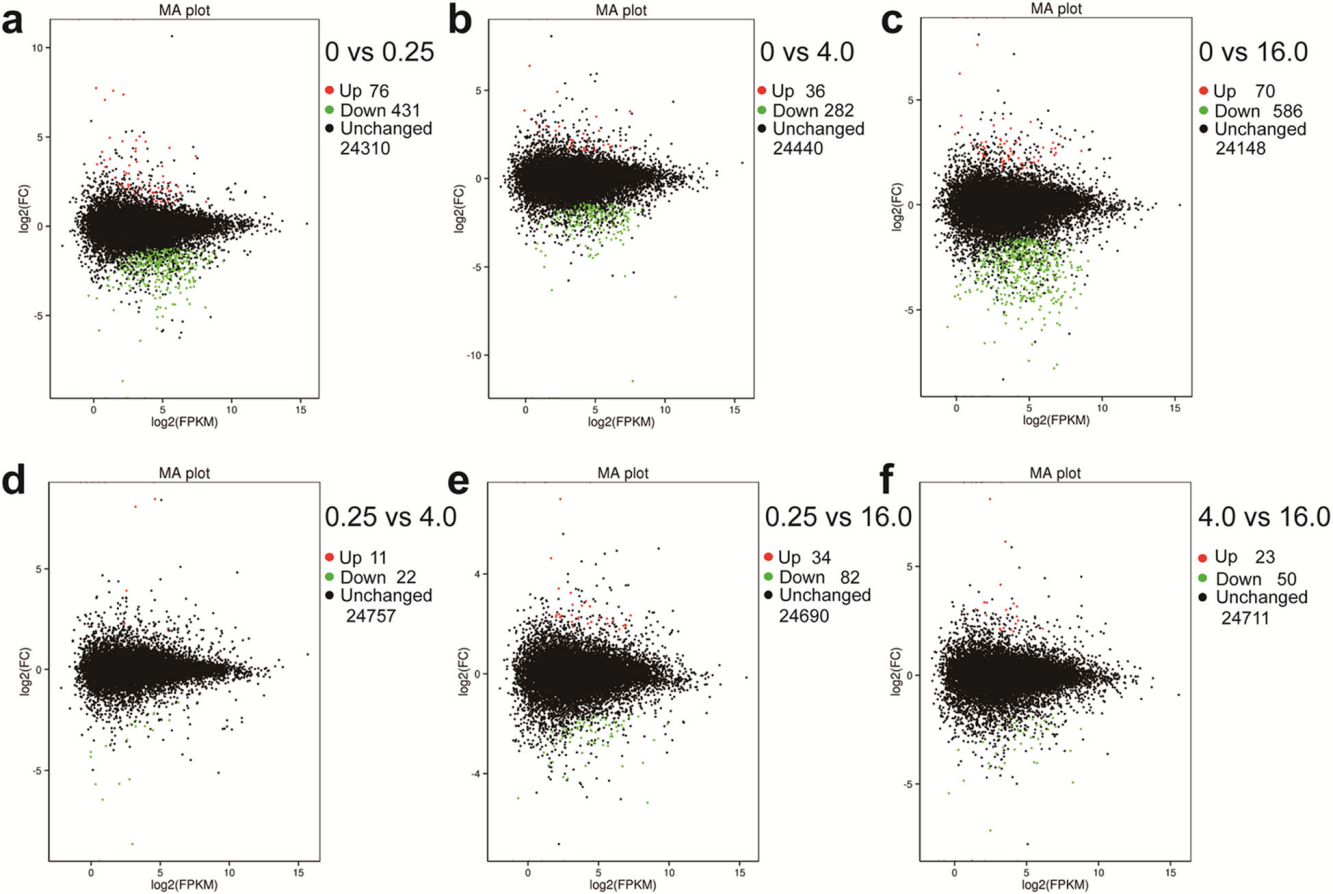
MA plots show the statistics of DEGs in the six comparison groups. **a**, 0 vs 0.25. **b**, 0 vs 4.0. **c**, 0 vs 16.0. **d**, 0.25 vs 4.0. **e**, 0.25 vs 16.0. **f**, 4.0 vs 16.0

The DEGs were filtered between six sample sets: 0 vs 0.25, 0 vs 4.0, 0 vs 16.0, 0.25 vs 4.0, 0.25 vs 16.0, and 4.0 vs 16.0. A total of 948 DEGs were obtained. The statistics of each comparison group showed the number of upregulated, downregulated, and unchanged transcripts in Fig. 4. The numbers of upregulated and downregulated transcripts were 76 and 431 (Fig. 4a), 36 and 282 (Fig. 4b), 70 and 586 (Fig. 4c), 11 and 22 (Fig. 4d), 34 and 82 (Fig. 4e), and 23 and 50 (Fig. 4f) in the 0 vs 0.25, 0 vs 4.0, 0 vs 16.0, 0.25 vs 4.0, 0.25 vs 16.0, and 4.0 vs 16.0 comparison group, respectively. The annotated numbers from the eight databases of the DEGs in each comparison group were counted (Table 4). The total numbers of the annotated DEGs and the percentages of the annotated DEG number in the total DEG number were 500 and 98.6%, 313 and 98.4%, 649 and 98.9%, 33 and 100%, 114 and 98.3%, and 73 and 100% in the 0 vs 0.25, 0 vs 4.0, 0 vs 16.0, 0.25 vs 4.0, 0.25 vs 16.0, and 4.0 vs 16.0 comparison groups, respectively. In addition, 11 transcripts involved in Se-metabolism were identified as DEGs, including F01_transcript/7937 (*Sultr1;1*), F01_transcript/8686 (*Sultr1;2*), F01_transcript/7028 (*Sultr2;1*), F01_transcript/17050 (*Sultr2;1*), F01_transcript/19508 (*APS2*), F01_transcript/21387 (*APR1*), F01_transcript/25148 (*APR1*), F01_transcript/26665 (*APR3*), F01_transcript/27008 (*APR3*), F01_transcript/28221 (*SBP1*), and F01_transcript/41258 (*SDI2*), which significantly enhanced expression under Na_2_SeO_4_ treatment compared to the control (Fig. 3).

**Table 4:** Statistics of the annotated DEGs

DEG_Set	Annotated	COG	GO	KEGG	KOG	Pfam	Swiss-Prot	eggNOG	Nr
0 vs 0.25	500	184	486	167	240	449	419	491	500
0 vs 4.0	313	122	306	120	157	290	261	303	312
0 vs 16.0	649	246	637	218	329	582	527	640	649
0.25 vs 4.0	33	14	33	13	19	31	30	33	33
0.25 vs 16.0	114	49	107	42	59	104	96	107	114
4.0 vs 16.0	73	35	70	38	43	72	64	69	73

**Fig. 5:**
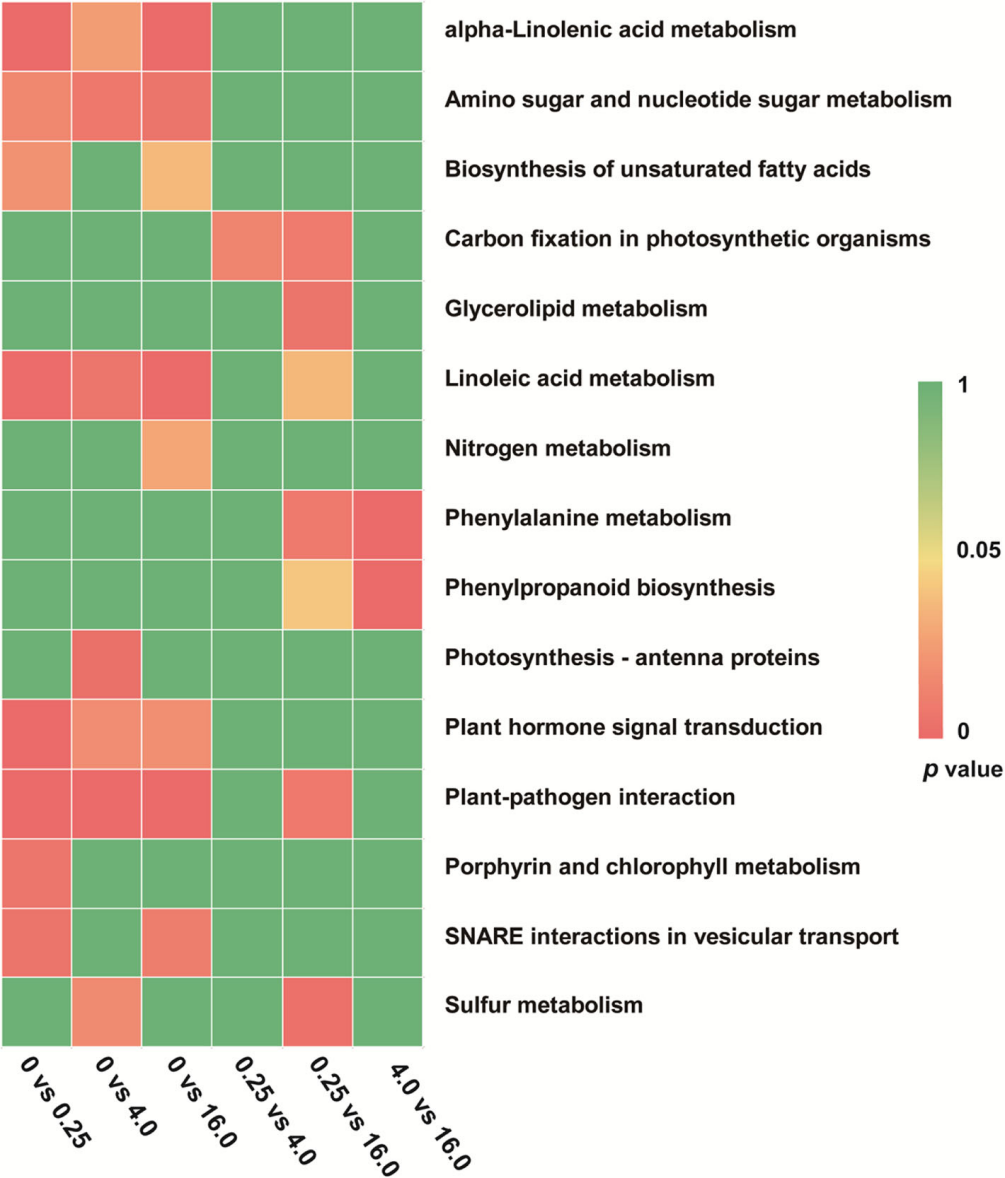
Enrichment of the top 15 KEGG pathways of the DEGs according to the *p* value. The red represents significant enrichment

The numbers of the annotated DEGs from KEGG were 167, 120, 218, 13, 42, and 38 in the 0 vs 0.25, 0 vs 4.0, 0 vs 16.0, 0.25 vs 4.0, 0.25 vs 16.0, and 4.0 vs 16.0 comparison groups, respectively. A total of 77 KEGG pathways were enriched from the six comparison groups (Additional file 8: Fig. S6). The 15 most significant KEGG pathways were displayed in Fig. 5. The alpha-linolenic acid metabolism (ko00592), amino sugar and nucleotide sugar metabolism (ko00520), linoleic acid metabolism (ko00591), plant hormone signal transduction (ko04075), and plant-pathogen interaction (ko04626) pathways were significantly enriched in 0 vs 0.25, 0 vs 4.0, and 0 vs 16.0 groups, indicating that these vital activities are related to Se metabolism in *C. violifolia*.

### Identification of single nucleotide polymorphism (SNP), simple sequence repeat (SSR), and alternative splicing (AS) events

**Fig. 6:**
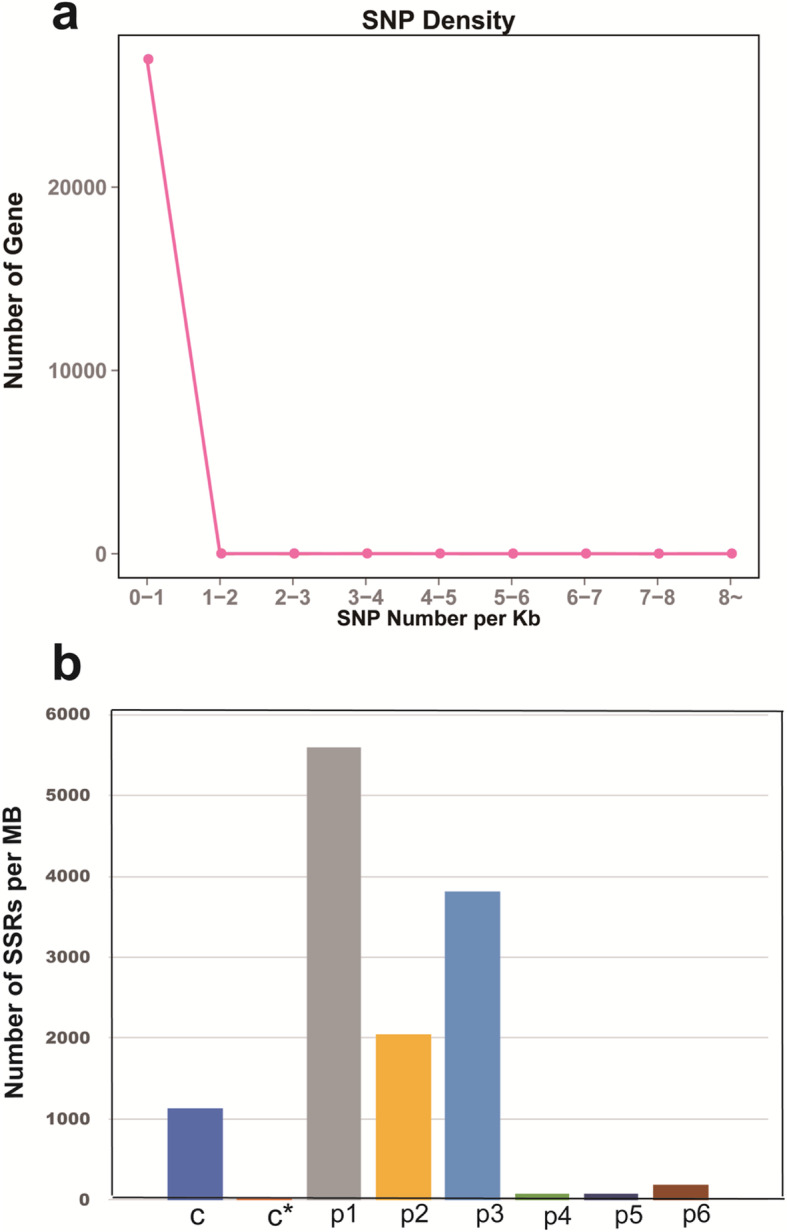
Density analysis of SNP and SSR. **a**, Density analysis of SNPs. **b**, Density analysis of the seven SSR types. c: compound SSR; p1: mononucleotide SSR; p2: dinucleotide SSR; p3: trinucleotide SSR; p4: tetranucleotide SSR; p5: pentanucleotide SSR; p6: hexanucleotide SSR

The transcripts in every sample were analyzed to identify SNP. Homozygotic (HomoSNP) and heterozygous (HeteSNP) SNPs were detected, and the total number of SNP in every sample was found to be approximately 180,000 (Additional file 9: Table S3). The analysis of SNP density showed that all the SNPs were distributed in 0–1 kb of the transcripts (Fig. 6a).

We examined 26,631 nonredundant sequences with a total size of 47,220,014 bp using the MIcroSAtellite (MISA) identification tool. Here obtained 14,269 SSRs. Analysis of SSR types showed that the numbers of compound, mononucleotide, dinucleotide, trinucleotide, tetranucleotide, pentanucleotide, and hexanucleotide SSRs were 1319, 6764, 2522, 4589, 94, 93, and 207, respectively (Additional file 10: Table S4). The density of mononucleotide SSRs was the highest, followed by those of trinucleotide SSR and dinucleotide SSRs (Fig. 6b).

The AS events of *C. violifolia* treated with selenate were predicted from the nonredundant transcripts using the BLAST software. Our data showed that 283 AS events were discovered in *C. violifolia*. To confirm the facticity of the predicting AS events, we randomly selected six candidate genes that were identified as AS events (The annotation of these six genes is as follows: F01_transcript/15968, uncharacterized Rho GTPase-activating protein At5g61530; F01_transcript/13543, heat shock 70 kDa protein 3; F01_transcript/18692, catalase-3; F01_transcript/2169, probable disease resistance protein At4g33300; F01_transcript/33954, phosphatidate cytidylyltransferase; F01_transcript/35375, transcription factor UNE12) for validation through reverse transcription PCR (RT-PCR). The results of RT-PCR confirmed the AS events of these six genes (Additional file 11: Fig. S7a-c). The three splicing isoforms of the six genes including F01_transcript/15968, F01_transcript/33954, and F01_transcript/35375 were remarkably differentially expressed in the four treatment groups. Na_2_SeO_4_ treatment seemed to enhance the expression of the isoforms. For example, the FPKM values of these three genes under 16 mg L^− 1^ Na_2_SeO_4_ treatment was 2.98, 1.64, and 1.58-fold of those in the 0 mg L^− 1^ Na_2_SeO_4_ treatment group, thereby indicating that the AS events play roles in Se accumulation in *C. violifolia*.

Also, we noted that one transcript identified as Se-metabolism related gene was alternatively spliced. RT-PCR validation confirmed the AS events of this gene (Additional file 12: Fig. S7d). That is F01_transcript/19477, annotated as *MMT*, which has been demonstrated to play a role in SeMet metabolism [8], indicating that metabolism of Se in *C. violifolia* may be regulated by AS.

### Prediction of TFs

A total of 3407 TFs categorized into 197 TF families were predicted from the nonredundant transcripts. The top 20 TF families with the most numbers of transcripts were analyzed (Additional file 12: Fig. S8). Among the top 20 TF families, bHLH contained the largest number of transcripts, followed by C3H and CAMK_CDPK. Several common TFs, such as MYB, WRKY, and C2H2, were also included in the top 20 TF families.

### Identification of long noncoding RNAs (lncRNAs)

**Fig. 7:**
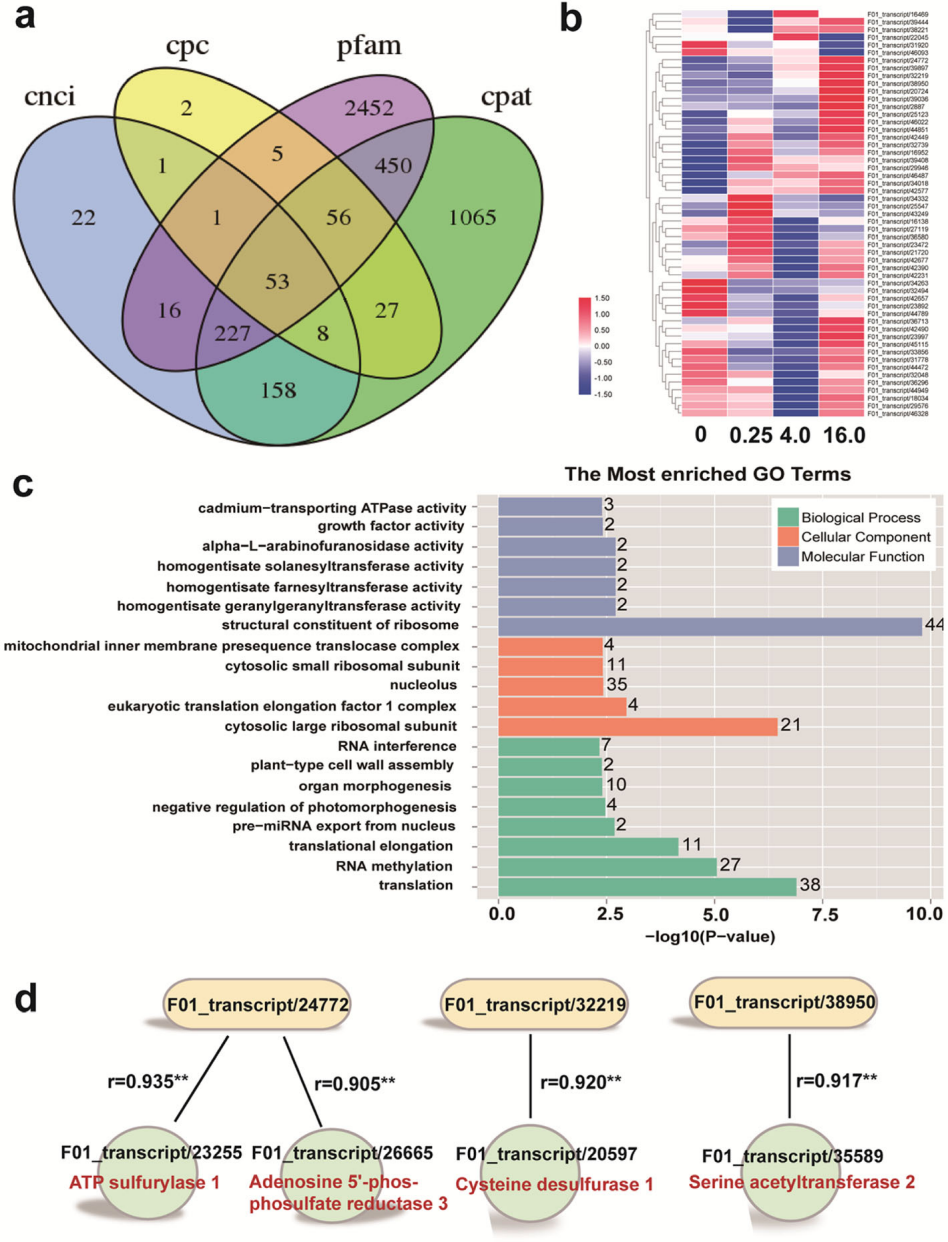
Analysis of lncRNA and the targeted genes. **a**, Venn diagram of lncRNA numbers from the four databases including CNCI, CPC, Pfam, and CPAT. **b**, Expression profiling of the predicted lncRNAs. **c**, GO enrichment analysis of the targeted genes of the lncRNAs. **d**, The screened targeted genes involved in Se metabolism and the corresponding lncRNAs

In plants, lncRNAs play important roles in gene expression regulation and response to external environmental stimulation [21, 22]. In this study, lncRNAs were predicted by analyzing the coding potential of the transcripts, using four methods, namely, coding potential calculator (CPC), coding-noncoding index (CNCI), coding potential assessment tool (CPAT), and Pfam. A total of 4543 lncRNAs were predicted from the above four databases, and the overlap (53 lncRNAs) of these four databases was further analyzed (Fig. 7a). Changes in the expression levels of the 53 lncRNAs in *C. violifolia* treated by selenate are shown in Fig. 7b. Among the 53 lncRNAs, 18 enhanced expression under different concentrations of Na_2_SeO_4_ treatment comparing with the 0 mg L^− 1^ Na_2_SeO_4_ treatment. The target genes of the 53 lncRNAs were also predicted, and a total of 797 target genes were obtained. Analysis of GO enrichment showed that these lncRNA targeted genes are significantly enriched in the structural constituent of ribosome, cytosolic large ribosomal subunit, and translation (Fig. 7c). The targeted genes contained four Se metabolism-related genes that are regulated by three lncRNAs (Fig. 7d). In detail, F01_transcript/23255 (*APS1*) and F01_transcript/26665 (*APR3*) are targeted by lncRNA F01_transcript/2477; F01_transcript/20597 (*cysteine desulfurase 1*) is targeted by lncRNA F01_transcript/32219; and F01_transcript/35589 (*serine acetyltransferase 2* [*SAT2*]) is targeted by lncRNA F01_transcript/38950. These results indicate that Se metabolism in *C. violifolia* may be regulated by lncRNAs.

### Gene co-expression analysis of the se metabolism-related genes and the lncRNAs

**Fig. 8:**
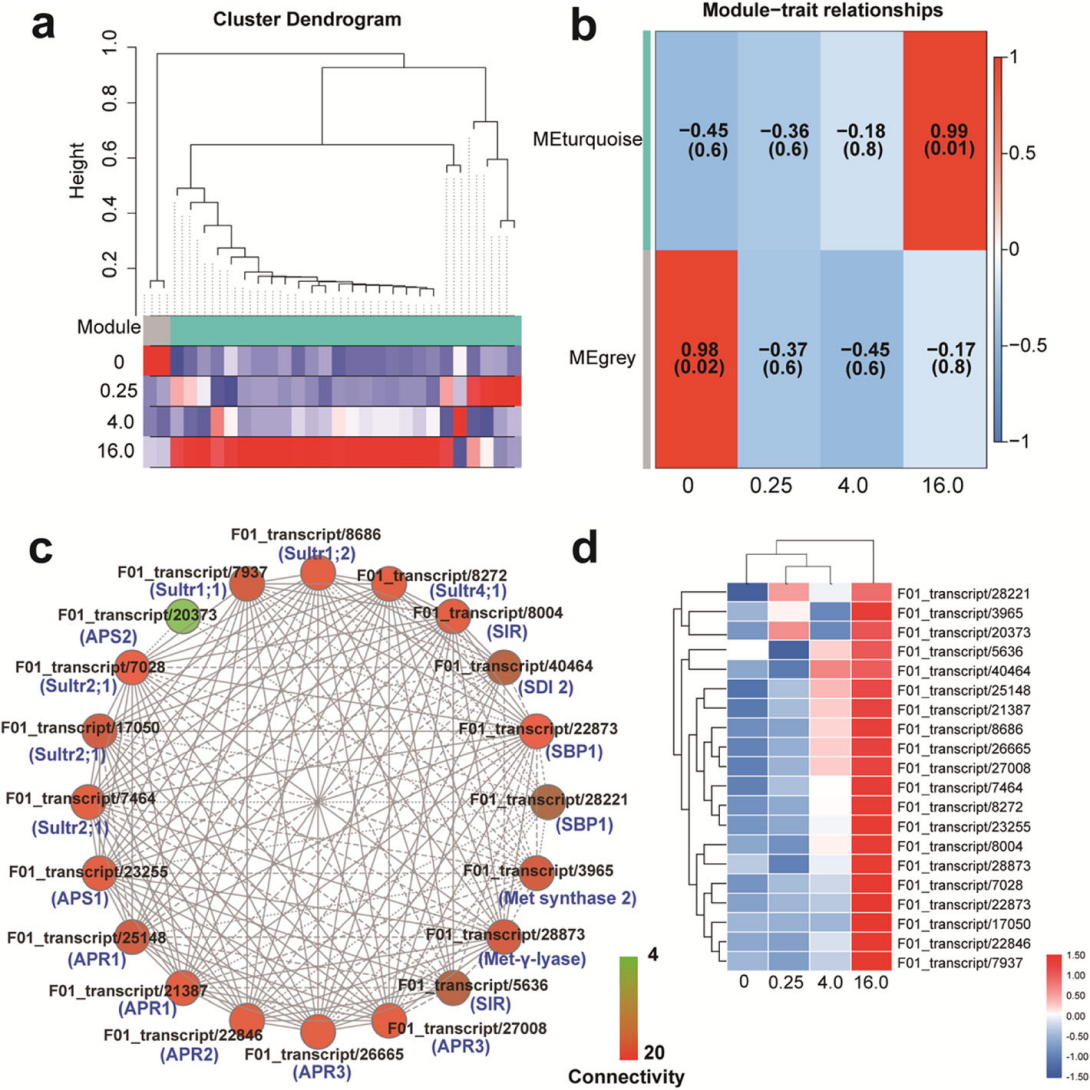
WGCNA analysis of the Se-related genes. **a**, Cluster dendrogram. **b**, Module-trait relationship analysis, the numbers inside the boxes were Pearson’s correlation coefficient and their *p*-value in the brackets. **c**, Co-expression network of the screened hub genes. **d**, Expression profile of the hub genes

According to the expression profiling of the 134 transcripts related to Se metabolism and the 53 lncRNAs in different samples, a weighted gene correlation network analysis (WGCNA) was performed to construct the gene co-expression modules for investigating the gene regulatory network of the Se hyperaccumulation of *C. violifolia* and screening key genes involved in its Se accumulation and tolerance of *C. violifolia*. The cluster dendrogram showed that the branches could be classified into two modules, that is, denoted in grey and turquoise (Fig. 8a). Module-trait relationship analysis showed that the turquoise module was significantly related to the 16.0 mg L^− 1^ Na_2_SeO_4_ treatment group (*r* = 0.99, *p* < 0.05) (Fig. 8b). A weighted network diagram was drawn to show the co-expression relationship and correlation between genes involved in the turquoise module (Fig. 8c). In the weighted network diagram, the degree of connectivity between genes is expressed by the color of the nodes. Red represents high connectivity and green represents low connectivity. The higher the connectivity, the more important the potential of the gene is in the hub of the regulatory network. As shown in Fig. 8c, 26 transcripts involved in the turquoise module were used to construct the regulatory network and 20 transcripts annotated in 14 genes were identified as candidate hub genes of the module, including four *Sultr* members, two *APS* members, three *APR* members, and one each of *SIR*, *Met synthase 2*, *MGL*, *SBP1*, and one gene encoding protein sulfur deficiency-induced 2 (*SDI2*). All the hub genes had high connectivity except F01_transcript/20373 (*APS2*), which implies that *APS2* plays a relatively weak role in the regulatory network. The expression levels of these 14 genes were enhanced by Na_2_SeO_4_ treatments, as the expression levels of these genes in the 16.0 mg L^− 1^ Na_2_SeO_4_ group were significantly increased than those in other treated samples (Fig. 8d), thereby indicating that these hub genes function as positive regulators in the Se accumulation and tolerance of *C. violifolia*. Specifically, the four transporter genes, namely *Sultr1;1; Sultr1;2*, *Sultr2;1*, and *Sultr4;1* were known to be involved in selenate uptake and translocation, the six assimilatory genes, namely, *APS1*, *APS2*, *APR1*, *APR2*, *APR3*, and *SIR* in selenate reduction and transformation, and the four specific genes, namely, *Met synthase 2*, *MGL*, *SBP1*, and *SDI2* in Se detoxification and tolerance, may play important roles in Se metabolism of *C. violifolia.*

### Correlation analysis of the key se metabolism-related genes and se concentration

Correlation analysis between *in planta* Se concentration and the 20 hub genes was executed (Table 5). The results indicate that all hub genes, except for F01_transcript/20373 (*APS2*)*,* F01_transcript/5636 (*SIR*), and F01_transcript/28221 (*SBP1*), were significantly correlated with the total, inorganic, and organic Se concentrations, especially F01_transcript/7937 (*Sultr1;1*), F01_transcript/23255 (*APS1*), F01_transcript/26665 (*APR3*), and F01_transcript/27008 (*APR3*), posed the highest correlations between their expressions and the total, inorganic, and organic Se concentrations, thereby again indicating that these genes play key roles in the Se accumulation and tolerance of *C. violifolia*.

**Table 5:** Correlation between the identified hub genes related to Se metabolism and the Se concentration (** means significant correlation at *p*<0.01, * means significant correlation at *p*<0.05)

Transcript ID	Total Se concentration	Inorganic Se concentration	Organic Se concentration
F01_transcript/7937 (*Sultr1;1*)	0.904**	0.874**	0.907**
F01_transcript/8686 (*Sultr1;2*)	0.705**	0.704**	0.704**
F01_transcript/7028 (*Sultr2;1*)	0.722**	0.696**	0.725**
F01_transcript/7464 (*Sultr2;1*)	0.523**	0.513**	0.524**
F01_transcript/17050 (*Sultr2;1*)	0.740**	0.710**	0.744**
F01_transcript/8272 (*Sultr4;1*)	0.873**	0.887**	0.870**
F01_transcript/23255 (*APS1*)	0.945**	0.945**	0.943**
F01_transcript/20373 (*APS2*)	0.276	0.257	0.278
F01_transcript/25148 (*APR1*)	0.891**	0.923**	0.895**
F01_transcript/21387 (*APR1*)	0.895**	0.905**	0.893**
F01_transcript/22846 (*APR2*)	0.788**	0.777**	0.789**
F01_transcript/26665 (*APR3*)	0.952**	0.965**	0.949**
F01_transcript/27008 (*APR3*)	0.972**	0.982**	0.969**
F01_transcript/5636 (*SIR*)	0.178	0.189	0.176
F01_transcript/8004 (*SIR*)	0.697**	0.734**	0.722**
F01_transcript/3965 (*Met synthase 2*)	0.419*	0.373*	0.426*
F01_transcript/28873 (*Met-γ-lyase*)	0.694**	0.690**	0.694**
F01_transcript/22873 (*SBP1*)	0.454*	0.442*	0.455*
F01_transcript/28221 (*SBP1*)	0.149	0.131	0.151
F01_transcript/40464 (*SDI2*)	0.414*	0.472*	0.414*

### Reliability analysis of the RNA-seq data

To validate the reliability of the RNA-seq data, we selected 51 transcripts associated with the Se metabolism-related genes for quantitative real-time PCR (qRT-PCR). The expression levels of these 51 transcripts were normalized to the expression of *β-actin*, *GAPB*, and *18S rRNA*. The results showed that the RNA-seq data (FPKM) and the qRT-PCR results (2^-ΔΔCt^) were significantly correlated (*R*^2^ = 0.7837, *p* < 0.01; Additional file 13: Fig. S9), indicating that the RNA-seq data are credible and accurate.

## Discussion

### PacBio FL transcriptome provided comprehensive gene information of *C. violifolia*

At present, few studies on the molecular mechanism of Se hyperaccumulation in *C. violifolia* are available. In particular, no reference genome of *C. violifolia* has yet been published, resulting in a lack of genetic information of this plant. These issues prevented the physiological and molecular mechanism of Se hyperaccumulation from elucidation. Here, we constructed a PacBio FL transcriptome database of *C. violifolia* treated with selenate and combined the Illumina RNA-seq data to mine genes involved in Se accumulation and tolerance in *C. violifolia* for the first time. This study yielded a total of 27,034 nonredundant transcripts, of which 26,745 were successfully annotated. Illumina RNA-seq revealed that 948 transcripts were differentially expressed from the four treatments of Na_2_SeO_4_. Enrichment analysis of KEGG pathway showed that the DEGs were significantly enriched in alpha-linolenic acid metabolism (ko00592), amino sugar and nucleotide sugar metabolism (ko00520), linoleic acid metabolism (ko00591), plant hormone signal transduction (ko04075), and plant-pathogen interaction (ko04626) pathways. Moreover, a total of 283 AS events and 14,269 SSRs were predicted using PacBio SMRT-seq technology. Specifically, 51 candidate genes and 53 lncRNAs as well as their predicted target genes we screened to be involved in Se metabolism. Weighted gene correlation network analysis (WGCNA) revealed that 20 hub genes might play important roles in Se metabolism and tolerance in *C. violifolia*. Therefore, the results of this study provided us comprehensive information into the genes involved in Se metabolism of *C. violifolia*.

### Candidate genes play roles in se metabolism and tolerance in *C. violifolia*

In this study, a total of 51 genes with 134 transcripts were identified as Se accumulation- and tolerance-related genes in *C. violifolia*. According to the gene annotation, we divided these genes into three classes, namely, transporter, assimilatory enzyme, and specific genes. Given the chemical similarity of Se and S, Se is metabolized via the S assimilatory pathway in plants [8]. Eight *Sultrs* genes, including *Sultr1;1*, *Sultr1;2*, *Sultr2;1*, *Sultr2;2*, *Sultr3;2*; *Sultr3;3*, *Sultr3;5*, and *Sultr4;1*, were found in *C. violifolia* (Additional file 7: Table S2). Among these genes, *Sultr1* is responsible for sulfate or selenate uptake in plants [23, 24]; *Sultr2* functions in sulfate translocation into the vascular system [25]; *Sultr3* plays important roles in S redistribution in plants, especially in S entering chloroplasts, and helps regulate the expression of Sultr2 [11, 26]; and *Sultr4* is responsible for sulfate vacuolar efflux [27]. Results of the comparative transcriptome revealed that the expression levels of all the *Sultrs* above were increased under Na_2_SeO_4_ treatments compared with the control group (Fig. 3), indicating that these genes may function in Se uptake and translocation in selenate-treated *C. violifolia*.

Thirty-one genes encoding assimilatory enzymes were screened from the FL transcriptome (Additional file 7: Table S2). Among these genes, *APSs* with four isoforms (*APS1*, *APS2*, *APS3*, and *APS4*) were identified. APS catalyzes selenate or sulfate by coupling ATP to form adenosine 5′-phosphoselenate (APSe) or adenosine 5′-phosphosulfate [6, 28, 29]. APSe can be further reduced to selenite by the catalyzation of APR [30], which was also identified in this study to have six isoforms (*APR1, APR2, APR3, APR4, APR5,* and *APR7*). Also, we identified two *adenylyl-sulfate kinases* (*ASKs*) genes, namely*, ASK1* and *ASK2*, and a *phosphoadenosine phosphosulfate* (PAPS) *reductase family protein*. During sulfate assimilation, adenosine 5′-phosphosulfate can be catalyzed by ASK to form PAPS [31] and is further converted to selenite under the mediation of PAPS reductase [32, 33]. However, the PAPS can also be assimilated to other secondary sulfated compounds in plants, such as glucosinolates [34]. The fate of sulfate in plants implies that selenate may be activated by ASK and PAPS reductase to form selenite or Se-containing S analogs, such as Se-containing glucosinolates [31–34]. Results of RNA-seq showed that the expression levels of these genes except *APR7* were significantly higher under Na_2_SeO_4_ treatments than those of the control, indicating that these genes may participate in the assimilation of selenate in *C. violifolia*. In sum, these genes related to Se assimilation obtained from our comprehensive transcriptome analysis provided essential genomic resources for studying the uptake and metabolism of Se in *C. violifolia*. Nevertheless, the catalytic functions of these genes in *C. violifolia* need to be confirmed by biochemical studies.

Sulfite can be reduced to sulfide under the mediation of SIR [35]. Sulfide can be applied to the formation of cysteine (Cys), which is synthesized by the action of Cys synthase (CS) and serine acetyltransferase (SAT) [36]. Given that *putative inactive CS 2* and *bifunctional L-3-cyanoalanine synthase/CS C1* possess the function of Cys synthesis [37–39], they may also play roles in SeCys synthesis. Hence, the activities of the two CS isoforms may complement to that of CS. As the homolog of SMT, HMT transforms homocysteine into Met in plants [40]. However, HMT has a high sequence identity with SMT, thereby indicating it has a similar function of methylating Cys and homocysteine [6]. In the present study, the genes responsible for the transformation of SeCys to SeMet, including *cystathionine gamma-synthase* (*CGS*) [41] and *Met synthase* [8] were found in (Additional file 7: Table S2). The expression levels of *CGS* and *Met synthase* were enhanced under Na_2_SeO_4_ treatments (Fig. 3). Therefore, it can be speculated that SeCys could be converted to MeSeCys or SeMet through the action of enzymes encoded by *HMT*, *CGS*, and *Met synthase* in *C. violifolia*. This process is potentially regulated by *S-adenosylmethionine synthetase* (*SAM*) in *C. violifolia* because *SAM* is the negative regulator of *CGS* [42]. Furthermore, SeMet could be further methylated to Se-methyl-SeMet under the mediation of *MMT* in *C. violifolia* according to integrated information from previous reports [9, 43].

Several specific genes that may be involved in Se metabolism in *C. violifolia* were identified (Additional file 7: Table S2, number 40–51) based on the results of studies on Se or S metabolism-related genes [8, 9]. Cys desulfurase degrades Cys and generates free S in plants [44]. Free S is utilized to form Fe–S clusters [45]. Here, several Fe–S protein genes were identified from the transcriptome data (e.g., *NADH dehydrogenase [ubiquinone] Fe–S protein 1 and Fe–S assembly protein IscA*). However, Cys desulfurase has an analogous enzyme, called SL, which can break down SeCys into elemental Se [18]. This result implies that the SeCys in *C. violifolia* may also be degraded into elemental Se under the activity of Cys desulfurase. Another S-containing aa, Met, can be degraded by the catalysis of MGL to generate methanethiol and 2-ketobutyrate [46], indicating that SeMet can be broken down into non-protein seleno-thiols. This speculation is indirectly supported by the study of Both et al. [1] that elemental Se accounts for 16% of total Se and SeMet is detected only in traces in *C. violifolia.* Besides, Met content is regulated by *Met over-accumulator* [47], whether SeMet is positively or negatively regulated by this gene is difficult to assess*.*

In Se hyperaccumulators, the high uptake of Se would mimic S deficiency due to the competitive roles of these two elements [48]. *SDI2* is a key negative regulator of glucosinolate synthesis in *Arabidopsis* under S-deficiency condition [49]. The enhancement of *SDI2* expression would lead to the depression of S-rich glucosinolates synthesis, thus making S usage to be prioritized for primary metabolites in plants under S-deficiency condition [49, 50]. Therefore, it is an important consideration to determine the S level in plants. The Se/S ratio level in tissues represents the Se accumulation capacity of plants [51]. *SBP1* is tightly related to detoxification when plants were under oxidative stress [52]. In Arabidopsis, overexpression of *SBP1* increased cadmium accumulation and reduced sensitivity to cadmium [53]. Evidence also shows that the expression of *SBP1* enhances the selenate tolerance of Arabidopsis by decreasing the sensitivity of stress [54]. Analysis of transcripts expression heatmap indicated that the expression levels of *SDI2* and *SBP1* were significantly increased with the concentration increase of Na_2_SeO_4_ treatment (Fig. 3 and Fig. 8d). Taken together, the transcription of *SDI2* and *SBP1* is likely to contribute to Se accumulation and tolerance of *C. violifolia*.

Here, WGCNA was performed to find the modules of highly correlated genes and summarized the intramodular hub genes related to the sample traits [55]. A total of 20 hub genes were identified and found to constitute a complex regulatory network (Fig. 8c). The biomass and physiological data showed that the *C. violifolia* plants grew normally under 0.25 and 4.0 mg L^− 1^ Na_2_SeO_4_ treatments, but were inhibited under 16.0 mg L^− 1^ Na_2_SeO_4_. However, the expression levels of these hub genes were significantly enhanced by 4.0 and 16.0 mg L^− 1^ selenate treatments (Fig. 8d), indicating that these hub genes can respond to selenate. Correlation analysis showed that these hub genes (except F01_transcript/20373 [*APS2*], F01_transcript/5636 [*SIR*], and F01_transcript/28221 [*SBP1*]) were significantly correlated with Se concentration (Table 5). Combining the functional analysis of these genes from previous studies on other Se hyperaccumulators, we suggest these genes are contributing to Se accumulation and tolerance in *C. violifolia*.

### Three lncRNAs may function in se metabolism in *C. violifolia*

Although Cakir et al. [56] demonstrated that several small noncoding RNAs could regulate Se accumulation in the hyperaccumulator *Astragalus chrysochlorus*, little information is available for lncRNA related to Se metabolism in plants. In the present study, 53 lncRNAs were predicted from the four databases (Fig. 7a) and the expression profiling showed expression levels of 18 lncRNAs were upregulated with the increase of selenate concentration, including F01_transcript/24772, F01_transcript/32219, and F01_transcript/38950. We identified the targeted transcripts of the 53 predicting lncRNAs (Fig. 7c). The targeted transcripts were remarkably enriched in ribosome and translation, which indicates that the translation process is regulated by these lncRNAs. This result may imply that Se detoxification is correlated with lncRNAs in *C. violifolia*. Also, three lncRNAs (F01_transcript/24772, F01_transcript/32219, and F01_transcript/38950) with four targeted transcripts involved in Se metabolism were identified using correlation analysis between the expression of lncRNAs and potential target genes (Fig. 7d). The four targeted Se-related transcripts are annotated as *APS1*, *APR3*, *Cys desulfurase 1*, and *SAT2*, and their expression levels were significantly enhanced (Fig. 3), thus indicating that the transcriptions of these genes are regulated by the corresponding lncRNAs. Since *APS1* and *APR3* play important roles in selenate reduction [28, 30], *Cys desulfurase 1* may function in transformation of SeCys to elemental Se [18], and *SAT2* is a key gene involved in SeCys synthesis [36], these three lncRNAs may influence the Se accumulation and tolerance of *C. violifolia* through regulating the expressions of the four genes.

### Putative se metabolism and assimilation pathway of *C. violifolia*

**Fig. 9:**
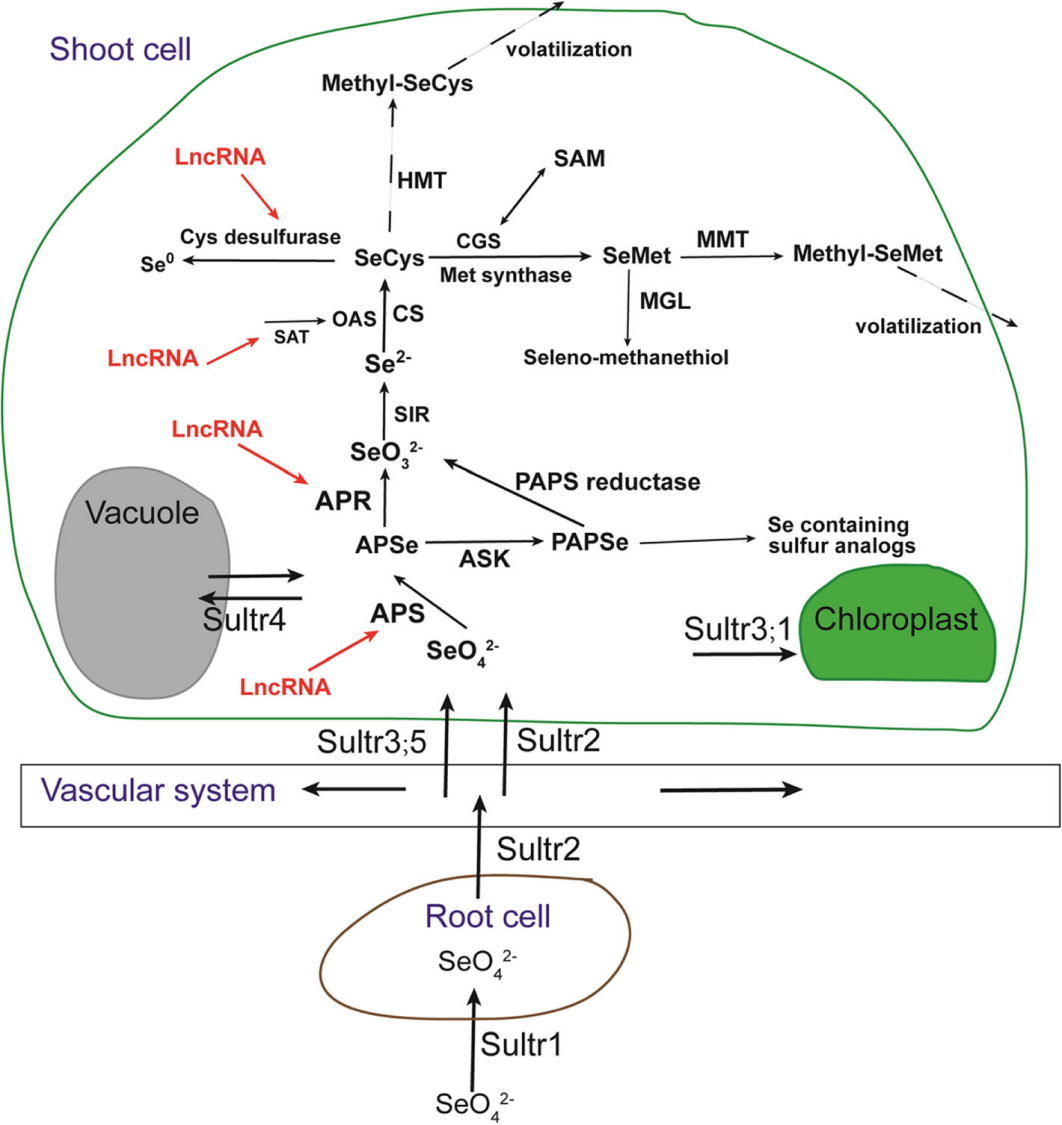
The putative Se metabolism pathway in *C. violifolia*. APR: adenosine 5′-phosphosulfate reductase, APS: adenosine triphosphate sulfurase, APSe: adenosine 5′-phosphoselenate, ASK: adenylyl-sulfate kinase, CGS: cystathionine gamma-synthase, CS: cysteine synthase, Cys: cysteine, HMT: homocysteine S-methyltransferase, MGL: methionine gamma-lyase, MMT: S-adenosyl-L-Met:Met-S-methyltransferase, PAPS: phosphoadenosine phosphosulfate, PAPSe: phosphoadenosine phosphoselenate, SAM: S-adenosylmethionine synthetase, SAT: serine acetyltransferase, SeCys: selenocysteine, SeMet: selenomethionine, SIR: selenite reductase, Sultr: sulfate transporter

Together with the results of the present study and gene characterization of previous work, we propose a putative Se metabolism and assimilation pathway in *C. violifolia* (Fig. 9). Selenate may be imported into root cells by Sultr1, and translocated to shoots via the vascular system by Sultr2 and Sultr3;5. Part of selenate can pass in and out the vacuoles under the mediation of Sultr4; some selenate may also enter chloroplasts under the mediation of Sultr3;1. Assimilation of selenate mainly takes place in the chloroplast and cytoplasm [8]. First, selenate is transformed into APSe by APS. APSe can be either catalyzed into selenite by APR or transformed into the intermediate compound PAPSe by the activity of ASK. PAPSe is further converted to other Se-containing S analogs or reduced into selenite by PAPS reductase. Selenite is reduced to selenide by SIR, and synthesized into SeCys by the combining actions of SAT, OAS, and CS. SeCys can be assimilated in three ways, namely, degradation of SeCys into elemental Se, which is mediated by Cys desulfurase; methylation of SeCys into MeSeCys by the enzyme HMT, where MeSeCys is eventually converted into volatile compounds [57]; and transformation of SeCys into SeMet by the activity of CGS and Met synthase. Also, SeMet can be degraded. This process is catalyzed by MGL and produces seleno-methanethiol. SeMet can also be methylated to MeSeMet under the catalyzation of MMT and then further volatilized. During the assimilation process, several genes are regulated by certain lncRNAs, such as *APS* and *APR*. Given that *MGL* and *Met synthase* were identified as hub genes, the formation and transformation of SeMet may be the main pathway through which Se is metabolized in *C. violifolia*. Looking forward to the future study, *Cys desulfurase* and *MGL* will be the key candidates for gene functional verification, because they are related to the detoxification of selenium in *C. violifolia*.

## Conclusion

The FL transcriptome of *C. violifolia* treated by selenate was analyzed, and a total of 26,745 annotated nonredundant transcripts were obtained. AS events, SSR, and SNP were also predicted to expand our genetic knowledge of *C. violifolia*. A total of 134 transcripts annotated in 51 genes were identified to be putatively involved in Se metabolism. Moreover, DEGs analysis showed that 11 Se-metabolism transcripts were differentially expressed by Na_2_SeO_4_ treatments. Several Se metabolism-related genes were predicted to be regulated by lncRNAs, which indicates that the latter may function in Se accumulation and tolerance in *C. violifolia*. Several hub genes were revealed by WGCNA, such as *Sultr1;1*, *SBP1*, and *MGL*. These hub genes may play key roles in Se accumulation and tolerance in *C. violifolia*. Finally, we proposed a putative Se metabolism pathway of *C. violifolia*. This study provides new insights into Se hyperaccumulation and tolerance in plants.

## Methods

### Plant materials

The seeds of *C. violifolia* were provided by Enshi Se-Run Health Tech Development Co., Ltd. (Enshi, China) and germinated in hole trays containing seedling substrate (pH 5.5, fiber length 0–6 cm, Pindstrup Co., Denmark). When the seedlings had grown four to five true leaves, they were dug out, rinsed twice, and transplanted into opaque hydroponics tanks for water culture. Each hydroponic tank measured of 36 cm in length, 24 cm in width, and 12.5 cm in depth, and featured 11 holes and an aerator. Immediately after transplantation, ^1^/_4_ Hoagland’s nutrient solution was used to acclimatize the plants to the water culture. The solution was replaced with ^1^/_2_ Hoagland’s nutrient solution after 10 days and added with Na_2_SeO_4_. Before the formal experiment, a preliminary experiment was performed to determine the Na_2_SeO_4_ concentrations of treatments, which would adopt *C. violifolia* for more than one month, rather than a short time response. Therefore, the treatments of Na_2_SeO_4_ concentrations in the nutrient solution were set at 0, 0.25, 4.0, and 16.0 mg L^− 1^. Each treatment had three independent biological replicates with eleven seedlings of *C. violifolia* plants in each replicate. The mixture of ^1^/_2_ Hoagland’s nutrient solution and sodium selenate was renewed every 10 days. The growth conditions of *C. violifolia* were controlled to a temperature of 20 ± 1 °C, relative humidity of 75%, irradiance of 200 μmol m^− 2^ s^− 1^, and 14 h photoperiod. The goal of this study is to find genes involved in Se metabolism and tolerance in *C. violifolia.* To fit this goal and avoid the errors between organs, the whole seedlings of *C. violifolia* from all replicates were harvested after 30 days of treatment, washed twice, frozen in liquid nitrogen, and stored at − 80 °C for Se concentration and transcriptome analysis.

### Determination of se, chlorophyll, free amino acid, and vitamin C content

Samples of whole seedlings of *C. violifolia* for Se concentration determination were dried at 60 °C to a constant weight and then ground into powder. Hydride generation atomic fluorescence spectrometry (HG-AFS) (AF640, Beifen-Ruili, Beijing, China) was used to determine the total Se concentration following the method of Yuan et al. (2013) [2] with minor modification. Briefly, 0.5 g samples were weighed and digested with 10 mL HNO_3_ and 2 mL H_2_O_2_ in a Microwave Digestion System (YMW40, Changsha Yong Le Kang Instrument, China). After digestion, 5 mL HCl was added into the digested solutions. The solutions were then continuously heated till cleared and transferred to 10 mL flasks, diluted with water to the set volume for determination. The HG-AFS conditions as follows: negative high voltage 340 V, lamp current 100 mA, temperature of atomization 800 °C, a flow rate of carrier gas 500 mL min^− 1^, injection volume 1 mL, KBH_4_ concentration 3.5%. The inorganic Se concentration, including SeO_3_^2−^ and SeO_4_^2−^, was determined via liquid chromatography–HG-AFS (LC-AFS8510, Beijing Haiguang Instrument, China) [2, 58]. The standard substances of SeO_3_^2−^ and SeO_4_^2−^ were purchased from the National Institute of Metrology, China. The determining conditions of SeO_3_^2−^ and SeO_4_^2−^ in the samples are as follows: mobile phase 40 mmol L^− 1^ KH_2_PO_4_ + 20 mmol L^− 1^ KCl, pH 6.0, flow rate 1.0 mL, chromatographic column Hamilton PRP-X100 (Hamilton Co., USA), column temperature 25 °C, injection volume 100 μL, cathodic current 80 mA, a flow rate of carrier gas 600 mL min^− 1^, negative high voltage 400 V. The organic Se concentration (including elemental Se) of *C. violifolia* was calculated as the difference between total Se and inorganic Se. Volatile Se species were not in the focus of the study, therefore their quantification was not carried out. Experiments were performed with three independent biological triplicates and each triplicate consisted of three technical replicates of Se determination.

Chlorophyll, free amino acid, and vitamin C contents were determined using ethanol extraction, ninhydrin colorimetry, and 2,6-dichloroindophenol titration method according to the methods recorded in ‘Experiments in Plant Physiology’ [59].

### Library preparation for Illumina RNA-seq and PacBio Iso-Seq sequencing

To construct PacBio Iso-Seq library, the total RNA of the whole *C. violifolia* seedlings was mixed from all the treatments of four Se concentrations in an equal amount that included the samples of 12 independent biological replicates. FL cDNA was synthesized by using the Clontech SMARter PCR cDNA Synthesis Kit (Mountain View, CA, USA) and filtered using BluePippin (Sage Science Beverly, MA, USA). One single-molecular real-time library of different lengths was constructed at Biomarker Tech. Co. (Beijing, China) using the Template Prep Kit 1.0 (PacBio, Menlo Park, CA, USA). Raw reads were processed into error-corrected reads of inserts (ROIs) using the Iso-seq pipeline with minFullPass = 0 and minPredictedAccuracy = 0.90. Then, FLNC transcripts were determined by searching for the polyA tail signal and the 5′ and 3′ cDNA primers in the ROIs. Iterative clustering for error correction (ICE) was used to obtain consensus isoforms and FL consensus sequences from ICE were polished by Quiver. High-quality FL transcripts were classified with a criteria post-correction accuracy of above 99%. Finally, redundancies were removed to obtain the final set of FL transcripts.

For the construction of Illumina RNA-seq libraries, total RNA of the whole *C. violifolia* seedling was extracted from four treatment groups of Se concentrations (0, 0.25, 4.0, and 16.0 mg L^− 1^). Three biological replications were set for each Se treated sample, and 12 independent cDNA libraries were constructed using the RNAprep Pure Plant Kit (Tiangen Biotech, Beijing, China). Illumina sequencing was performed by using the Illumina HiSeq 2500 platform at Biomarker Tech. Co. (Beijing, China). Clean reads were obtained by removing the raw reads containing adapter, poly-N, and low-quality reads. The clean reads were then mapped to the PacBio reference sequence. Only reads with a perfect match or only one mismatch were further analyzed and annotated based on the reference sequence. Gene expression levels are expressed as fragments per kilobase of transcript per million fragments mapped (FPKM). FPKM from the 12 independent samples was used for statistical analysis. The EBSeq R package was used to screen DEGs by comparing two samples. Transcripts with False Discovery Rates < 0.05 and |log2(fold change)| ≥ 1 were screened as DEGs. Enrichment analysis of DEGs was analyzed using the KEGG databases to obtain the pathway enrichment of the DEGs.

### Transcript function annotation and identification of transcripts related to se metabolism

BLAST software (version 2.2.26) was applied to annotate the nonredundant transcripts by searching against several databases, including Nr, KEGG, Pfam, KOG, COG, egg-NOG, swiss-Prot, and GO. The GOseq package in R was used to analyze GO enrichment, and KOBAS software was used to analyze KEGG enrichment. Transcripts involved in Se metabolism were screened by searching the integrative annotation results.

### Identification of AS events and RT-PCR validation

AS events were predicted via the pairwise alignment of all nonredundancy FL transcripts using BLAST software. Candidate AS events were filtered to meet the following conditions: the length of the two AS isoforms should be longer than 1000 bp with two high-scoring segments pairs, the gap between AS should be longer than 100 bp, the distant between AS and the 3′/5′ end should be at least 100 bp, and a 5 bp overlap should be allowed in all AS. The primers used for RT-PCR were designed in Primer3Plus (http://www.primer3plus.com/cgi-bin/dev/primer3plus.cgi) (Additional file 14: Table S5) to validate AS events according to the method of Ye et al. [60].

### Identification of lncRNA and targeting transcript prediction

Coding RNA candidates from the set of putative protein-coding RNAs in the transcripts were sorted by combining four computational approaches, including the CPC, CNCI, CPAT, and Pfam database. Putative protein-coding RNAs were filtered out using a minimum length and exon number threshold. Transcripts with lengths of greater than 200 nt and more than two exons were selected as lncRNA candidates. CPC, CNCI, CPAT, and Pfam were further used to screen the lncRNAs. The overlapping result of these four approaches was applied for the subsequent analysis. The targeting transcripts of lncRNAs were performed by analyzing the correlation between lncRNA and mRNA expression [61].

### WGCNA analysis

The screened transcripts related to Se metabolism and the lncRNAs obtained were used to construct co-expression networks via the R package WGCNA (version 1.42). FPKM values of the transcripts from the 12 independent samples belonging to the four treatment groups were used for WGCNA. Modules were obtained using the automatic network construction function blockwiseModules with the default settings. Weighted network diagram was drawn using the OmicShare tools, a free online platform for data analysis (http://www.omicshare.com/tools).

### Coding sequence detection and identification of TF

TransDecoder software (https://github.com/TransDecoder/TransDecoder/releases) was applied to identify the reliable coding sequences (CDS) based on the length of the ORF, log-likelihood score, aa sequence, and alignment information of the protein domain structures obtained from the Pfam database [60]. TFs were predicted by using the iTAK software [62].

### SSR analysis

Transcripts longer than 500 bp were screened for SSR analysis using MISA software (http://pgrc.ipk-gatersleben.de/misa/) [63]. The identified SSRs were classified into seven types, namely, mononucleotide, dinucleotide, trinucleotide, tetranucleotide, pentanucleotide, hexanucleotide, and compound SSRs.

### SNP analysis

Picard-tools (version 1.41) and Samtools (version 0.1.18) were used to sort, remove duplicated reads, and merge the BAM alignment results of each sample. GATK software was used to perform SNP calling [64]. Raw vcf files were filtered by using the GATK standard filter method and other parameters (e.g., clusterWindowSize: 10, MQ0 ≥ 4, [MQ0/{1 × DP}] > 0.1, QUAL< 10; QUA< 30 or QD < 5 or HRun> 5]), and only SNPs with a distance of > 5 were retained. SNPs could be divided into HomoSNP and HeteSNP according to the number of alleles in the SNP sites.

### Gene expression via qRT-PCR

Each cDNA of mRNA was amplified by real-time PCR using the AceQ Universal SYBR qPCR Master Mix and the HiScript III-RT SuperMix for qPCR (+gDNA wiper) (Vazyme Biotech, Nanjing). The LineGene 9600 Plus Fluorescent Quantitative PCR System (Bioer, Hangzhou, China) was used for qRT-PCR analysis. The relative expressions of all tested transcripts were normalized to the reference gene *β-actin 3, GAPB (a GAPDH family member),* and *ribosomal 18 s rRNA,* and calculated using the formula F = 2^-ΔΔCt^ [65]. The primers for qRT-PCR were designed by using Primer3Plus (Additional file 15: Table S6).

### Statistical analysis

Data from all the treatments were expressed as mean values representing three biological triplicates ± standard errors. Data were analyzed using SPSS22 (SPSS Inc., Chicago, IL, USA) by one-way ANOVA. Comparisons between multiple treatment groups were performed by Tukey’s honestly significant difference test at *p* ≤ 0.05.

## Supplementary information

**Additional file 1: Fig. S1.** Quality assessment of the RNA extracted from each Na_2_SeO_4_ treated group.

**Additional file 2: Fig. S2.** Length distribution of the consensus reads.

**Additional file 3: Fig. S3.** Hierarchical cluster analysis of all the nonredundant transcripts.

**Additional file 4: Fig. S4.** Protein length distribution of the predicted coding sequences.

**Additional file 5: Table S1.** Integrity annotation of all the transcripts. (XLS 24495 kb)

**Additional file 6: Fig. S5.** Statistic analysis from COG and eggNOG database**. a**, COG annotation statistics. **b**, eggNOG annotation statistics.

**Additional file 7: Table S2.** The screened transcripts related to S/Se metabolism.

**Additional file 8: Fig. S6**. Significance analysis of all the 77 KEGG pathways enriched from the six comparison groups.

**Additional file 9: Table S3.** Statistics of SNP numbers in all samples.

**Additional file 10: Table S4.** Statistic of SSR numbers.

**Additional file 11: Fig. S7.** RT-PCR validation of AS events. **a**, Validation of F01_transcript/13543, F01_transcript/15968, F01_transcript/18692, and F01_transcript/2169. **b**, Validation of F01_transcript/33954. **c**, Validation of F01_transcript/35375.

**Additional file 12: Fig. S8.** Transcript number statistics of the top 20 transcription factors.

**Additional file 13: Fig. S9.** Correlation analysis of the results between RNA-seq and qRT-PCR.

**Additional file 14: Table S5.** Primers used for RT-PCR validation.

**Additional file 15: Table S6.** Primers used for qRT-PCR.

## Data Availability

The raw PacBio sequence data reported in this paper have been deposited in the Genome Sequence Archive in BIG Data Center [66], Beijing Institute of Genomics (BIG), Chinese Academy of Sciences, under accession number CRA002432 that is publicly accessible at https://bigd.big.ac.cn/gsa. All the raw RNA-seq data are available at the National Center for Biotechnology Information sequence read archive (accession NO. PRJNA590869). The database generated and the materials used during the current study are available from the corresponding and first authors on reasonable request (xufeng198@126.com; raoshen1989@163.com).

## Abbreviations

aa: Amino acid
AFS: Hydride generation atomic fluorescence spectrometry
APR: Adenosine 5′-phosphosulfate reductase
APS: Adenosine triphosphate sulfurase
APSe: Adenosine 5′-phosphoselenate
AS: Alternative splicing
ASK: Adenylyl-sulfate kinase
CCS: Circular consensus sequences
CDS: Coding sequences
CGS: Cystathionine gamma-synthase
COG: Clusters of Orthologous Groups
CNCI: Coding-non-coding index
CPAT: Coding potential assessment tool
CPC: Coding potential calculator
CS: Cysteine synthase
CSL: Cys sulfoxide lyase
Cys: Cysteine
DEG: Differently expressed gene
DMSe: Dimethyl-selenide
DMDSe: Dimethyl-diselenide
egg-NOG: Evolutionary genealogy of genes Nonsupervised Orthologous Groups
FL: Full-length
FLNC: Full-length non-chimeric
GO: Gene Ontology
HeteSNP: Heterozygous SNP
HG-AFS: Hydride generation atomic fluorescence spectrometry
HMT: Homocysteine S-methyltransferase
HomoSNP: Homozygotic SNP
KEGG: Kyoto Encyclopedia of Genes and Genomes
KOG: Eukaryotic Orthology Groups
lncRNA: Long non-coding RNAs
MeSeCys: Methyl selenocysteine
Met: Methionine
MGL: Methionine gamma-lyase
MISA: MIcroSAtellite identification tool
MMT: S-adenosyl-L-Met:Met-S-methyltransferase
NCBI: National Center for Biotechnology Information
NOG: Non-supervised Orthologous Groups
Nr: The NCBI non-redundant protein sequences
nt: Nucleotides
ORF: Open reading frames
PAPS: Phosphoadenosine phosphosulfate
PAPSe: Phosphoadenosine phosphoselenate
Pfam: Protein family
qRT-PCR: Quantitative real-time RCR
RT-PCR: Reverse transcription –PCR
SAM: S-adenosylmethionine synthetase
SAT: Serine acetyltransferase
SBP: Selenium binding protein
SDI: Protein sulfur deficiency-induced
SeCys: Selenocysteine
SeCys2: Selenocystine
SeMet: Selenomethionine
SIR: Selenite reductase
SL: SeCys lyase
SMRT: Single-molecule real-time
SMT: SeCys methyltransferase
SNP: Single nucleotide polymorphism
SSR: Simple sequence repeats
Sultr: Sulfate transporter
TF: Transcription factors
WGCNA: Weighted gene correlation network analysis
