# Supplementary figures and images for: Integration analysis of PacBio SMRT- and Illumina RNA-seq reveals candidate genes and pathway involved in selenium metabolism in hyperaccumulator Cardamine violifolia
Source: BMC Plant Biol. 2020 Oct 27;20:492. doi: 10.1186/s12870-020-02694-9 (PMC7590678; doi:10.1186/s12870-020-02694-9)

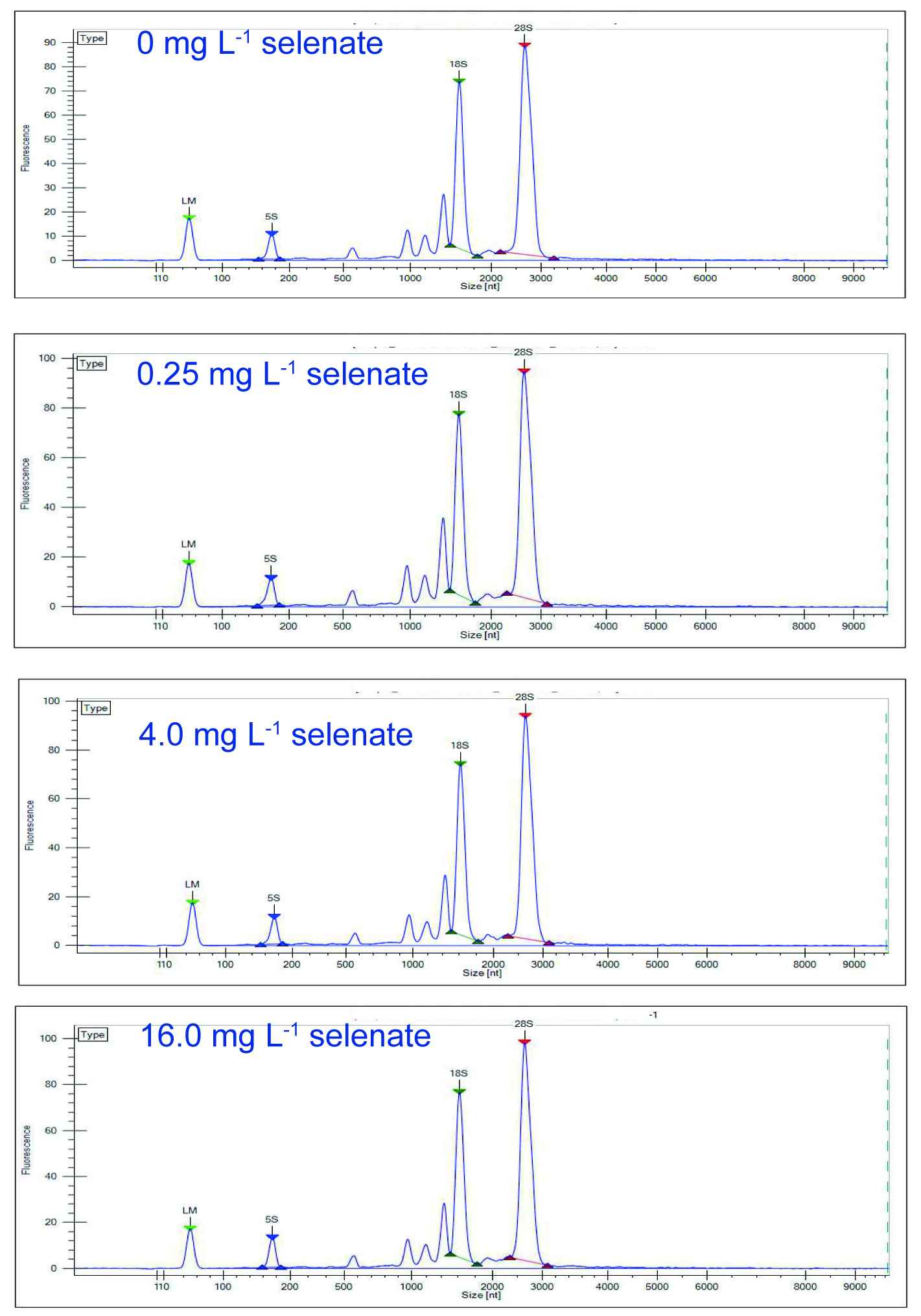

Supplement: Supplementary file 1 — Additional file 1: Fig. S1. Quality assessment of the RNA extracted from each Na2SeO4 treated group. [file 12870_2020_2694_MOESM1_ESM.tif]

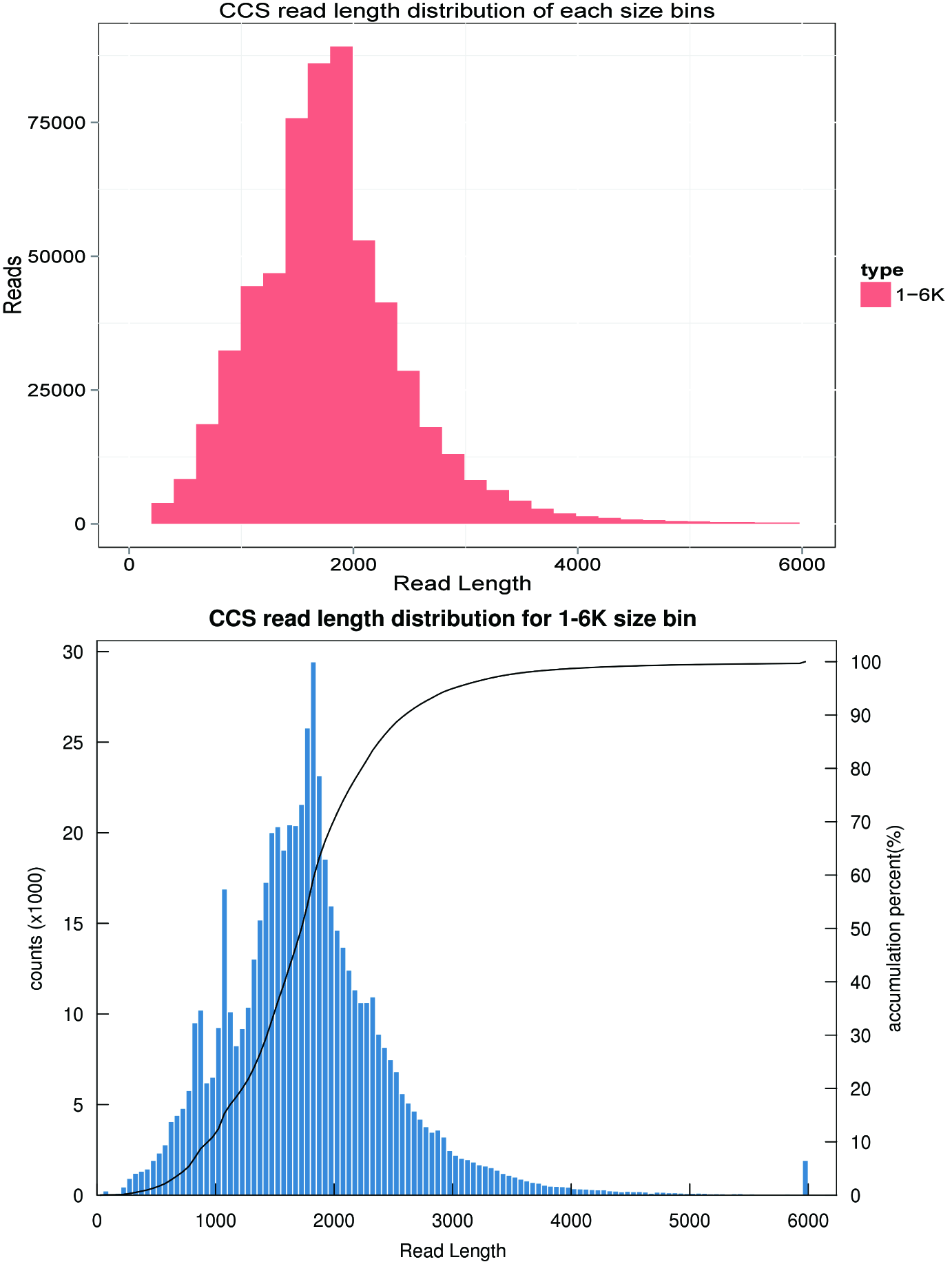

Supplement: Supplementary file 2 — Additional file 2: Fig. S2. Length distribution of the consensus reads. [file 12870_2020_2694_MOESM2_ESM.tif]

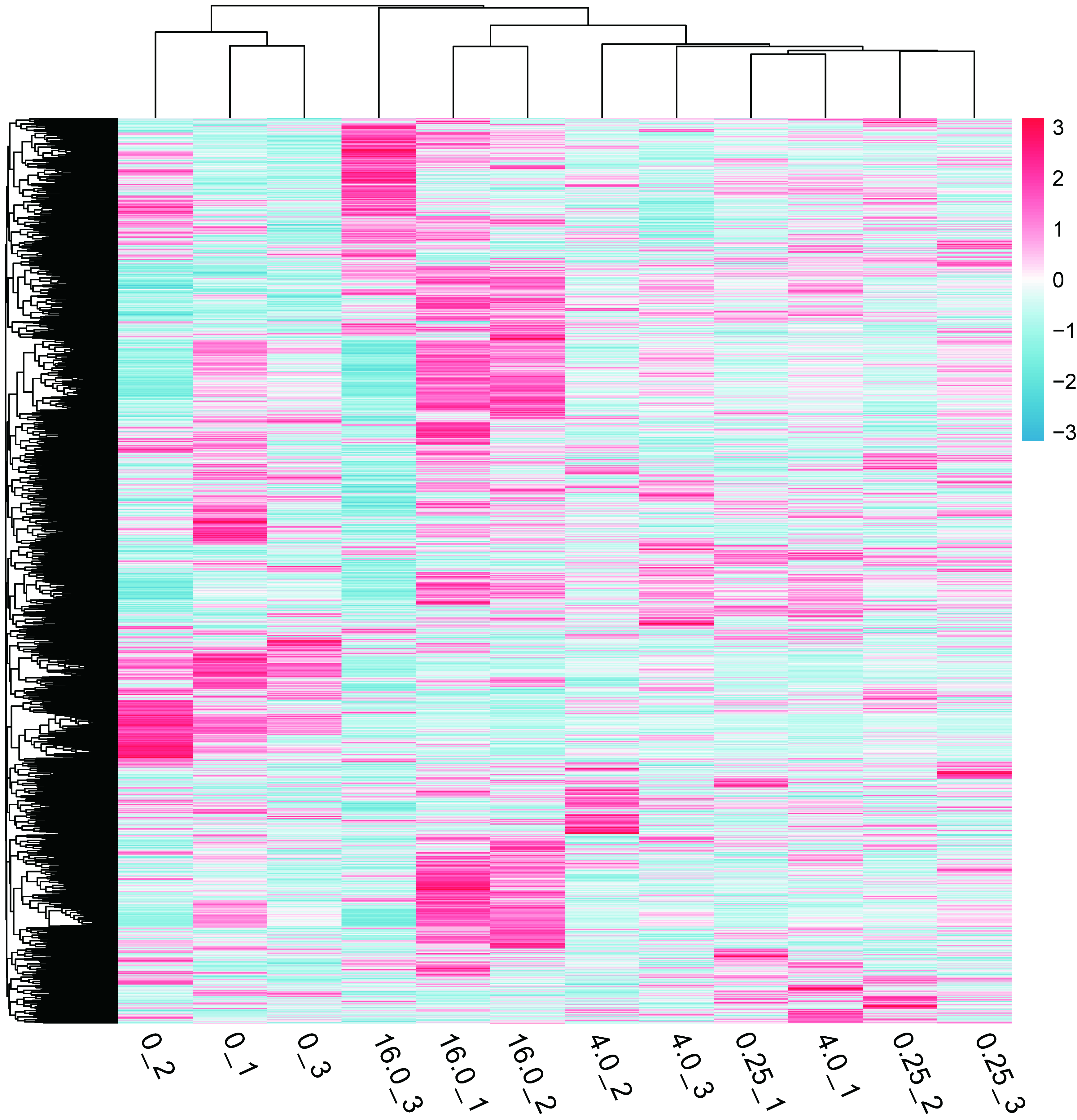

Supplement: Supplementary file 3 — Additional file 3: Fig. S3. Hierarchical cluster analysis of all the nonredundant transcripts. [file 12870_2020_2694_MOESM3_ESM.tif]

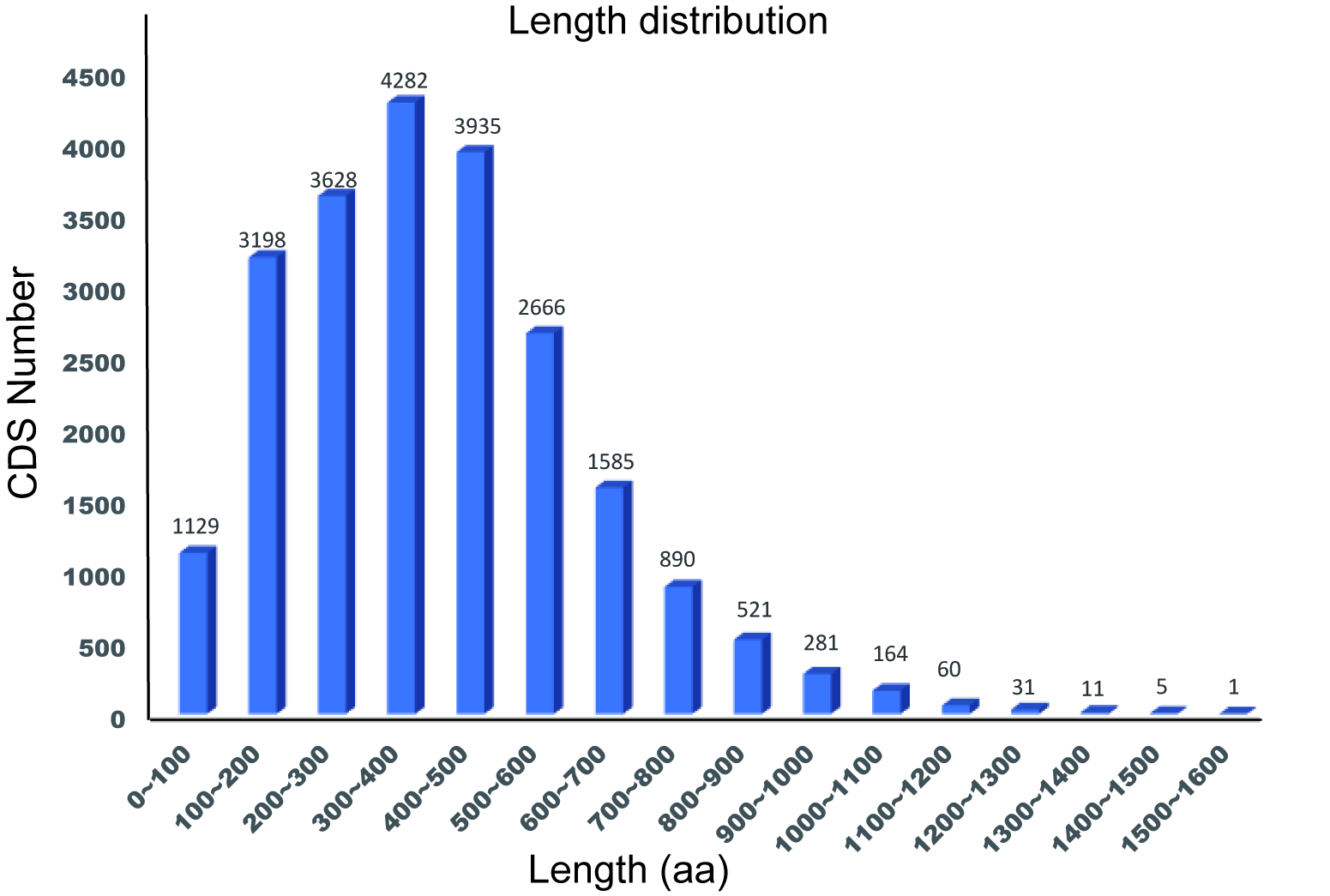

Supplement: Supplementary file 4 — Additional file 4: Fig. S4. Protein length distribution of the predicted coding sequences. [file 12870_2020_2694_MOESM4_ESM.tif]

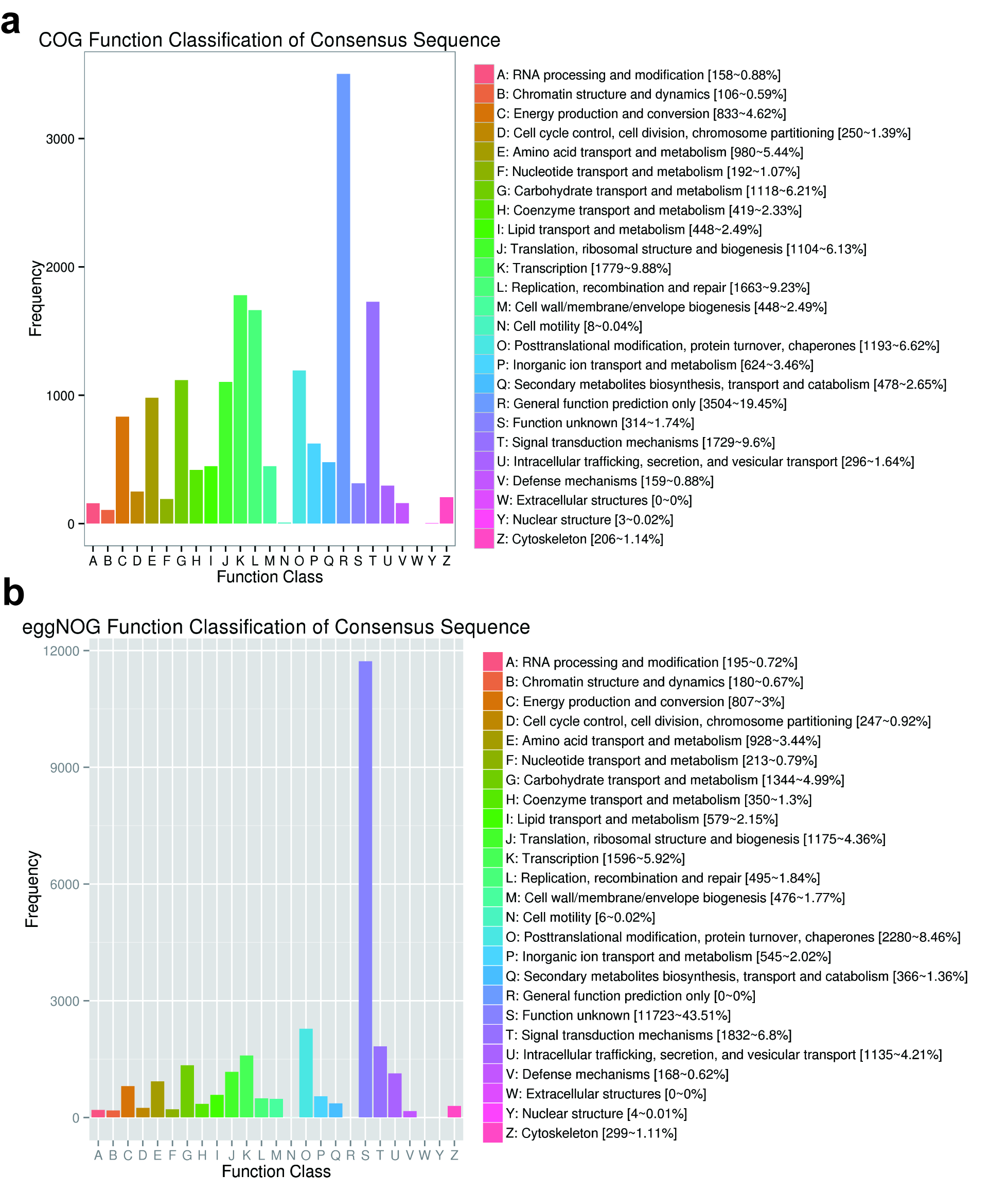

Supplement: Supplementary file 6 — Additional file 6: Fig. S5. Statistic analysis from COG and eggNOG database. a, COG annotation statistics. b, eggNOG annotation statistics. [file 12870_2020_2694_MOESM6_ESM.tif]

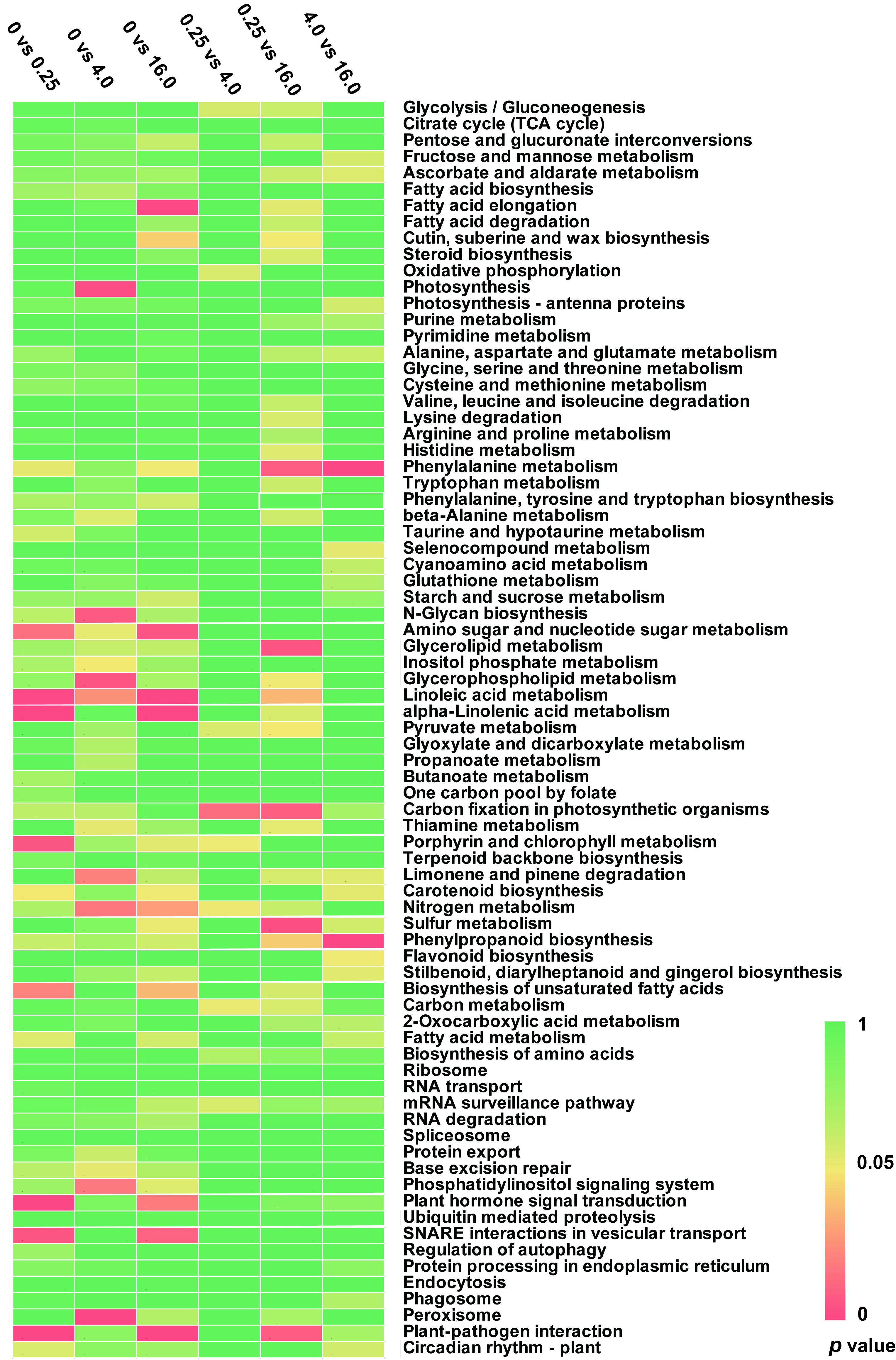

Supplement: Supplementary file 8 — Additional file 8: Fig. S6. Significance analysis of all the 77 KEGG pathways enriched from the six comparison groups. [file 12870_2020_2694_MOESM8_ESM.tif]

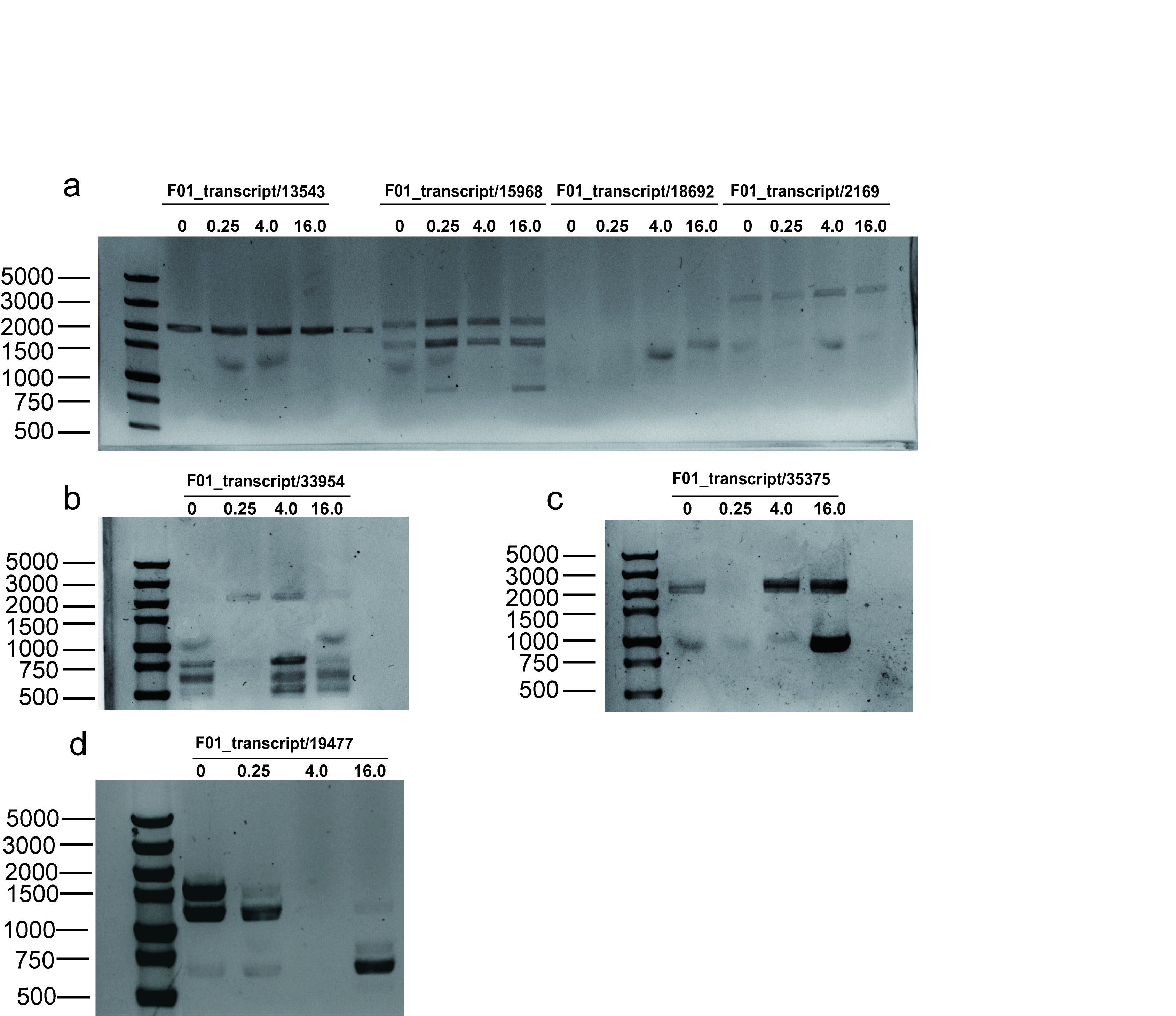

Supplement: Supplementary file 11 — Additional file 11: Fig. S7. RT-PCR validation of AS events. a, Validation of F01_transcript/13543, F01_transcript/15968, F01_transcript/18692, and F01_transcript/2169. b, Validation of F01_transcript/33954. c, Validation of F01_transcript/35375. [file 12870_2020_2694_MOESM11_ESM.tif]

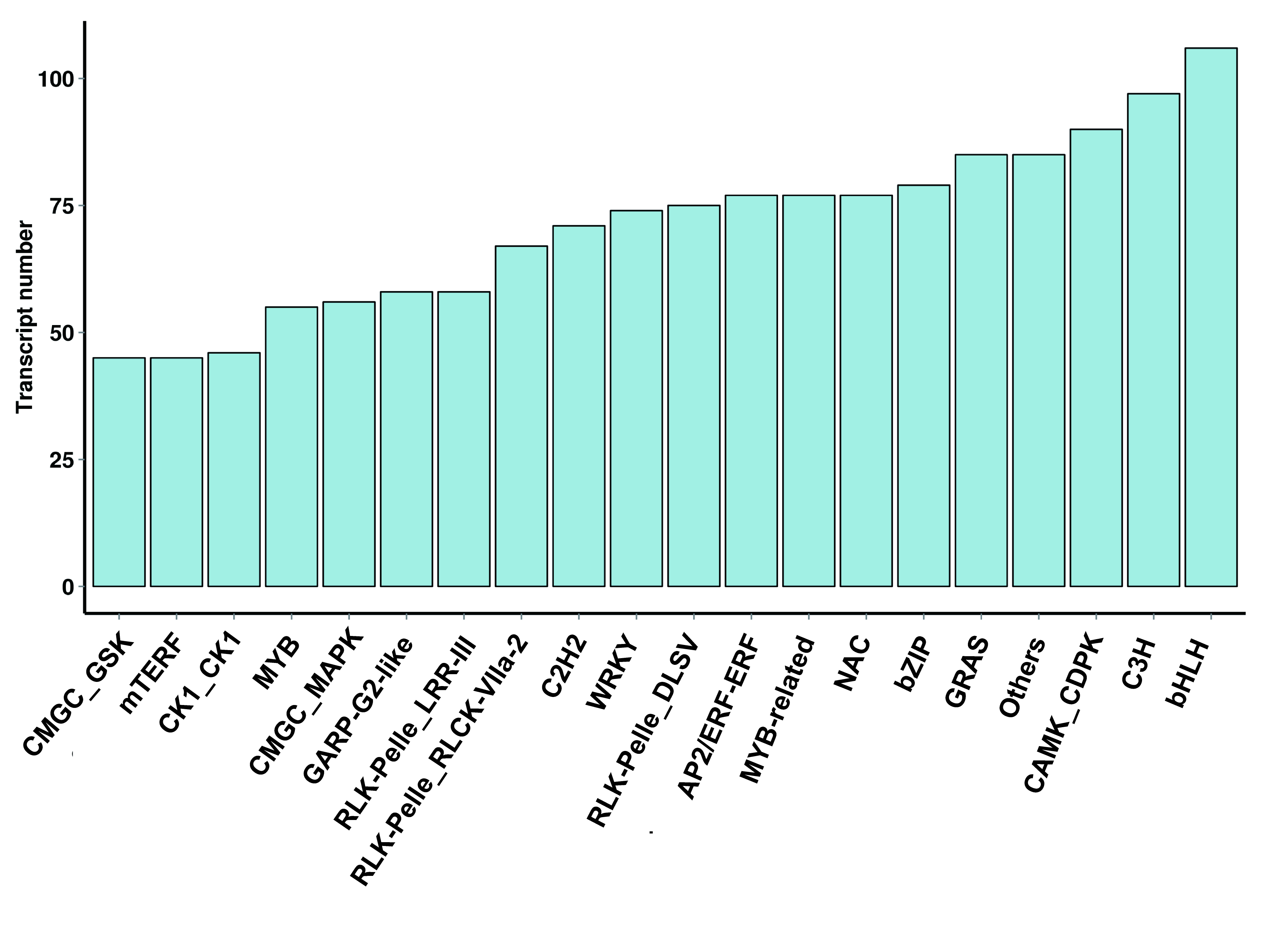

Supplement: Supplementary file 12 — Additional file 12: Fig. S8. Transcript number statistics of the top 20 transcription factors. [file 12870_2020_2694_MOESM12_ESM.tif]

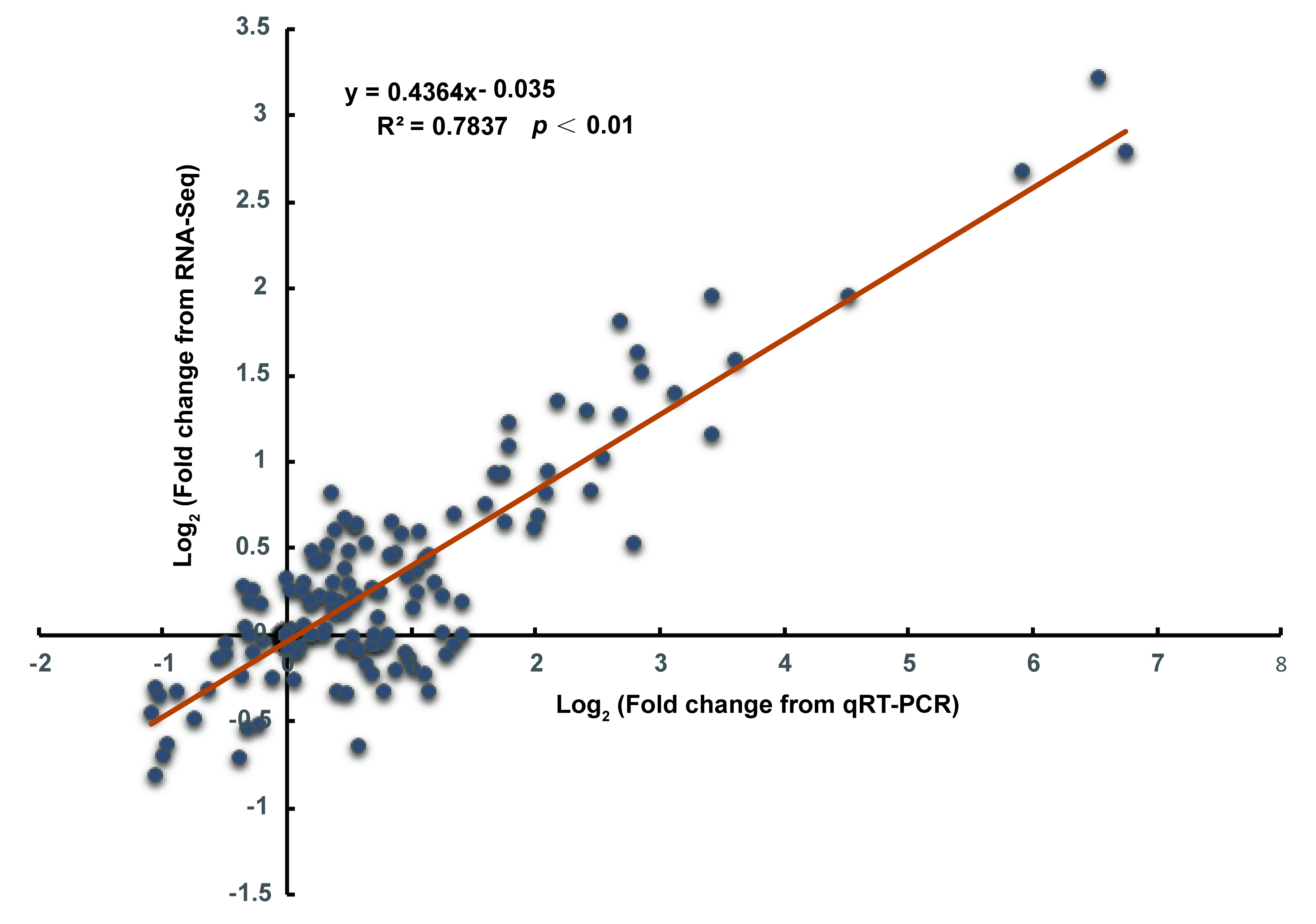

Supplement: Supplementary file 13 — Additional file 13: Fig. S9. Correlation analysis of the results between RNA-seq and qRT-PCR. [file 12870_2020_2694_MOESM13_ESM.tif]
