# Supplementary material for: Integration analysis of PacBio SMRT- and Illumina RNA-seq reveals candidate genes and pathway involved in selenium metabolism in hyperaccumulator Cardamine violifolia
Source: BMC Plant Biol. 2020 Oct 27;20:492. doi: 10.1186/s12870-020-02694-9 (PMC7590678; doi:10.1186/s12870-020-02694-9)
Supplement: Supplementary file 7 — Additional file 7: Table S2. The screened transcripts related to S/Se metabolism. [file 12870_2020_2694_MOESM7_ESM.docx]

**Table S2** The screened transcripts related to S/Se metabolism

|  | **Transcript ID** | | **Annotation** | | |
| --- | --- | --- | --- | --- | --- |
| 1 | F01_transcript/7937, F01_transcript/9356,  F01_transcript/8918, F01_transcript/7575 | | | Sulfate transporter 1.1 | |
| 2 | F01_transcript/8686, F01_transcript/13872 | | | Sulfate transporter 1.2 | |
| 3 | F01_transcript/7028, F01_transcript/7464, F01_transcript/10004, F01_transcript/17050 | | | Sulfate transporter 2.1 | |
| 4 | F01_transcript/11311, F01_transcript/11344, F01_transcript/14574 | | | Sulfate transporter 2.2 | |
| 5 | F01_transcript/9927 | | | Sulfate transporter 3.2 | |
| 6 | F01_transcript/10893, F01_transcript/31922, F01_transcript/8838 | | | Sulfate transporter 3.3 | |
| 7 | F01_transcript/21735 | | | Sulfate transporter 3.5 | |
| 8 | F01_transcript/8272 | | | Sulfate transporter 4.1 | |
| 9 | F01_transcript/20208, F01_transcript/23255, F01_transcript/50093, F01_transcript/21620 | | | ATP sulfurylase 1 | |
| 10 | F01_transcript/19508, F01_transcript/20606, F01_transcript/23365, F01_transcript/20373 | | | ATP sulfurylase 2 | |
| 11 | F01_transcript/19613, F01_transcript/22649 | | | ATP sulfurylase 3 | |
| 12 | F01_transcript/25371, F01_transcript/25671, F01_transcript/31173, F01_transcript/50804 | | | ATP sulfurylase 4 | |
| 13. | F01_transcript/40439, F01_transcript/42391 | | | Adenylyl-sulfate kinase 1 | |
| 14 | F01_transcript/39482 | | | Adenylyl-sulfate kinase 2 | |
| 15 | F01_transcript/21387, F01_transcript/25148 | | | adenosine 5'-phosphosulfate reductase 1 | |
| 16 | F01_transcript/22846 | | | adenosine 5'-phosphosulfate reductase 2 | |
| 17 | F01_transcript/26665, F01_transcript/27008 | | | adenosine 5'-phosphosulfate reductase 3 | |
| 18 | F01_transcript/34537 | | | adenosine 5'-phosphosulfate reductase 4 | |
| 19 | F01_transcript/29995, F01_transcript/33232, F01_transcript/33392 | | | adenosine 5'-phosphosulfate reductase 5 | |
| 20 | F01_transcript/36977, F01_transcript/37331, F01_transcript/38710, F01_transcript/39397, F01_transcript/42748 | | | adenosine 5'-phosphosulfate reductase 7 | |
| 21 | F01_transcript/13865, F01_transcript/16081,  F01_transcript/21691 | | | Phosphoadenosine phosphosulfate  reductase family protein (unnamed) | |
| 22 | F01_transcript/39143, F01_transcript/46027,  F01_transcript/5636, F01_transcript/8004,  F01_transcript/8621, F01_transcript/8683  F01_transcript/9957 | | | Sulfite reductase | |
| 23 | F01_transcript/42448 | | | Serine acetyltransferase 1 | |
| 24 | F01_transcript/35589 | | | Serine acetyltransferase 2 | |
| 25 | F01_transcript/30526, F01_transcript/44036 | | | Cysteine synthase | |
| 26 | F01_transcript/40680 | | | putative inactive cysteine synthase 2 | |
| 27 | F01_transcript/36762, F01_transcript/37274 | | | Bifunctional L-3-cyanoalanine synthase/cysteine synthase C1 | |
| 28 | F01_transcript/36857 | | | Homocysteine S-methyltransferase 1 | |
| 29 | F01_transcript/41611, F01_transcript/40466 | | | Homocysteine S-methyltransferase 2 | |
| 30 | F01_transcript/15640, F01_transcript/16185,  F01_transcript/43150 | | | Cystathionine gamma-synthase 1 | |
| 31 | F01_transcript/3965 | | | Methionine synthase 2 | |
| 32 | F01_transcript/3519 | | | Methionine synthase 3 | |
| 33 | F01_transcript/2729 | | | Cobalamin-independent  methionine synthase | |
| 34 | F01_transcript/26861, F01_transcript/29961,  F01_transcript/31746, F01_transcript/35743 | | | S-adenosylmethionine synthase | |
| 35 | F01_transcript/43371 | | | S-adenosylmethionine synthetase 1 | |
| 36 | | F01_transcript/32064, F01_transcript/35791,  F01_transcript/43680 | S-adenosylmethionine synthetase 2 | |  |
| 37 | | F01_transcript/18735, F01_transcript/21257, F01_transcript/26865, F01_transcript/27996,  F01_transcript/36442 | S-adenosylmethionine synthetase 3 | |  |

**Table S3** continued

| 38 | F01_transcript/405, F01_transcript/41367,  F01_transcript/32950, F01_transcript/43934,  F01_transcript/43819, F01_transcript/45989,  F01_transcript/15253, F01_transcript/16318,  F01_transcript/16515, F01_transcript/16998,  F01_transcript/19477, F01_transcript/28885,  F01_transcript/3297, F01_transcript/19753,  F01_transcript/3294, F01_transcript/41119,  F01_transcript/22943, F01_transcript/33333,  F01_transcript/42210, F01_transcript/5842 | S-adenosyl-L-methionine  -dependent methyltransferases  superfamily protein |
| --- | --- | --- |
| 39 | F01_transcript/38148, F01_transcript/38601 | 1. adenosyl-methionine-sterol-C   -methyltransferase 3 |
| 40 | F01_transcript/20137, F01_transcript/20597 | Cysteine desulfurase 1 |
| 41 | F01_transcript/21493, F01_transcript/24731,  F01_transcript/25231, F01_transcript/22110 | Cysteine desulfurase 2 |
| 42 | F01_transcript/3597, F01_transcript/3845,  F01_transcript/5853 | NADH dehydrogenase [ubiquinone]  iron-sulfur protein 1 |
| 43 | F01_transcript/50209 | NADH dehydrogenase [ubiquinone]  iron-sulfur protein 5-B |
| 44 | F01_transcript/44409, F01_transcript/47790 | NADH dehydrogenase [ubiquinone]  iron-sulfur protein 7 |
| 45 | F01_transcript/43592 | NADH dehydrogenase [ubiquinone]  iron-sulfur protein 8-B |
| 46 | F01_transcript/47920 | Iron-sulfur assembly protein IscA |
| 47 | F01_transcript/28873, F01_transcript/32169 | Methionine gamma-lyase |
| 48 | F01_transcript/20416 | Methionine over-accumulator |
| 49 | F01_transcript/18779 | Threonine synthase 1/methionine  over-accumulator |
| 50 | F01_transcript/41258 | Protein sulfur deficiency-induced 2 |
| 51 | F01_transcript/22873, F01_transcript/23953,  F01_transcript/28221, F01_transcript/32505 | Selenium-binding protein 1 |
