# Supplementary material for: Integration analysis of PacBio SMRT- and Illumina RNA-seq reveals candidate genes and pathway involved in selenium metabolism in hyperaccumulator Cardamine violifolia
Source: BMC Plant Biol. 2020 Oct 27;20:492. doi: 10.1186/s12870-020-02694-9 (PMC7590678; doi:10.1186/s12870-020-02694-9)
Supplement: Supplementary file 9 — Additional file 9: Table S3. Statistics of SNP numbers in all samples. [file 12870_2020_2694_MOESM9_ESM.docx]

**Table S3** The statistics of SNP number in all samples

| Treatments | Samples | HomoSNP | HeteSNP | All SNP |
| --- | --- | --- | --- | --- |
| 0 | repeat 1 | 111,701 | 69,425 | 181,126 |
|  | repeat 2 | 109,557 | 67,457 | 177,014 |
|  | repeat 3 | 105,768 | 74,315 | 180,083 |
| 0.25 | repeat 1 | 105,865 | 75,488 | 181,353 |
|  | repeat 2 | 107,966 | 69,870 | 177,836 |
|  | repeat 3 | 108,155 | 73,983 | 182,138 |
| 4.0 | repeat 1 | 104,314 | 76,922 | 181,236 |
|  | repeat 2 | 113,401 | 69,099 | 182,500 |
|  | repeat 3 | 106,086 | 74,560 | 180,646 |
| 16.0 | repeat 1 | 106,341 | 73,981 | 180,322 |
|  | repeat 2 | 111,554 | 69,388 | 180,942 |
|  | repeat 3 | 107,967 | 69,256 | 177,223 |

HomoSNP: homozygotic SNP, HeteSNP: heterozygous SNP.
