# Supplementary material for: Integration analysis of PacBio SMRT- and Illumina RNA-seq reveals candidate genes and pathway involved in selenium metabolism in hyperaccumulator Cardamine violifolia
Source: BMC Plant Biol. 2020 Oct 27;20:492. doi: 10.1186/s12870-020-02694-9 (PMC7590678; doi:10.1186/s12870-020-02694-9)
Supplement: Supplementary file 10 — Additional file 10: Table S4. Statistic of SSR numbers. [file 12870_2020_2694_MOESM10_ESM.docx]

**Table S4** The statistic of SSR number

| Searching item | Numbers |
| --- | --- |
| Total number of sequences examined | 26,631 |
| Total size of examined sequences (bp) | 47,220,014 |
| Total number of identified SSRs | 14,269 |
| Number of SSR containing sequences | 10,184 |
| Number of sequences containing more than 1 SSR | 2,944 |
| Number of SSRs present in compound formation | 1,319 |
| Mono-nucleotide | 6,764 |
| Di-nucleotide | 2,522 |
| Tri-nucleotide | 4,589 |
| Tetra-nucleotide | 94 |
| Penta-nucleotide | 93 |
| Hexa-nucleotide | 207 |
