# Supplementary material for: Integration analysis of PacBio SMRT- and Illumina RNA-seq reveals candidate genes and pathway involved in selenium metabolism in hyperaccumulator Cardamine violifolia
Source: BMC Plant Biol. 2020 Oct 27;20:492. doi: 10.1186/s12870-020-02694-9 (PMC7590678; doi:10.1186/s12870-020-02694-9)
Supplement: Supplementary file 14 — Additional file 14: Table S5. Primers used for RT-PCR validation. [file 12870_2020_2694_MOESM14_ESM.docx]

**Table S**5 The primers used for RT-PCR validation

| Gene ID | | F | R |
| --- | --- | --- | --- |
| F01_transcript/13543 | ATGGCTGGTAAAGGAGAAGG | | CTATGATCGGGTTGCAAAAC |
| F01_transcript/15968 | ATGCCTTCTCTCATATCACAAC | | TCATGGCCTCTTGTCTTCT |
| F01_transcript/2169 | ATGGCAATCACCGATTTTTTCG | | CTACTCGTCGAGCCAATCTAG |
| F01_transcript/33954 | ATTCTCGCGCATTAGTAACTG | | TCAACGGTGTTCTTCCAAAG |
| F01_transcript/35375 | ATGGCTAGTAACAACCCTCAC | | CTACGGTGGAGGATTCGTC |
| F01_transcript/18692 | ATGGATCCTTACAAGTATCGTCC | | TCAGAGATTGGTCTCTAGATGC |
| F01_transcript/19477 | GAGGGAAGAAGAAGAACCAG | | TTACTCCATCTGAAAATAGAGAATATGC |
