# Supplementary material for: Integration analysis of PacBio SMRT- and Illumina RNA-seq reveals candidate genes and pathway involved in selenium metabolism in hyperaccumulator Cardamine violifolia
Source: BMC Plant Biol. 2020 Oct 27;20:492. doi: 10.1186/s12870-020-02694-9 (PMC7590678; doi:10.1186/s12870-020-02694-9)
Supplement: Supplementary file 15 — Additional file 15: Table S6. Primers used for qRT-PCR. [file 12870_2020_2694_MOESM15_ESM.docx]

**Table S6** The primers used for qRT-PCR

| Gene ID | F | R |
| --- | --- | --- |
| F01_transcript/17050 | GAAGACGGTGAGGCACATCA | CGCAATCGCCTCGGTTAAAG |
| F01_transcript/7937 | AACTTTGTGTCAGGCCGTGA | CTCCTCCCATGAACCCAACC |
| F01_transcript/8686 | AACCATCGCAAGTCTCTGCA | GGATCGACCTCAGCTCGAAG |
| F01_transcript/21387 | GCCGTGTGAGAAAGGTGAGA | ATTCGCAACCGGATTCCACT |
| F01_transcript/27008 | TGGATCGTTGTGCTTTACGC | TGCAACCTTCACGCCATTTC |
| F01_transcript/28221 | GAGCCTCGATGGGAAACGAT | GAGCAAGTGCAGGACCTTCT |
| F01_transcript/41258 | GAGCCAGATGCAAACAAGGC | TCGGATCACCAGACCCTTCT |
| F01_transcript/22649 | AGGAACCCGGTTCATAATGGAC | TCGTAAACCCTCCCAATGGATG |
| F01_transcript/25371 | CACGCCAAAGCTAGGATCAATG | TCCCGTGATCTGCATCATACAG |
| F01_transcript/40439 | AAGGGTCTCTACAAGCTTGCTC | CCATTGGTGAAGTTCCGGTTTC |
| F01_transcript/39482 | AAGAATCGGTGAGGTGGCTAAG | ATCGCCCTCAGGTAACAAAGAC |
| F01_transcript/22846 | GCGGTTGTGCAAGAGATATTCG | CAACCATGGCTCTTTACGGTTC |
| F01_transcript/34537 | ACTATGCGGGCAAGATATCGAG | CCTTATCCAGCGTTTCAATGCC |
| F01_transcript/33392 | GTAACCTGATCACATGGTTGCG | GGAAGACGAGGATTGCCATTTG |
| F01_transcript/42748 | TCGAAGATGCGATACCATGGTC | CATCAGTGTCCAAGCTTGTTGG |
| F01_transcript/13865 | CTTGCTGCTACAAACACTGAGG | CTTGCTGCTACAAACACTGAGG |
| F01_transcript/8683 | CCAAACGTCAATGAGTCAGCTG | TCTTCCACCACGTTCTTCTCTG |
| F01_transcript/42448 | GACGATGTCTGGATCAAGATGC | TGAGCTTTACGGAGAGGATGTG |
| F01_transcript/35589 | GGCGGAAACTATTAGCTCTTGC | TTCTAGCCGCTGGATGTATGTC |
| F01_transcript/30526 | CAGTTTGACAACCCAGCCAATC | AATCGACCGACACCAGTGATAG |
| F01_transcript/40680 | TGAGCTGGTTCTTACTGATCCG | CTGCAGCATGTAACCATTAGGC |
| F01_transcript/37274 | CACATGCTATCACAGGCAATGG | ACGATATCCCAACCATGAGACC |
| F01_transcript/36857 | CTGGTGCAGACATTGTGGTTAC | TAGCAAAGACTCGCTCTCTTCC |
| F01_transcript/40466 | CAGGCATTTGCTGAGCTGTTAG | AATCCCCACTAACCACATTCCC |
| F01_transcript/16185 | GTTCATGCCGGTGAACGATTAG | AGGCAGATGTGTTGACTACAGG |
| F01_transcript/3965 | TTCCCCAAGAGTCACCAATGAG | TGGGAGGATTGGAAGGTTTAGC |
| F01_transcript/3519 | TCTCCTATGCATCTCACAAGGC | TAGGACCAATGAGCACAGGAAC |
| F01_transcript/2729 | AAGAGCAGTGCTGAGGATCTTC | ATGTACTTGGTCCCAGCATCAG |
| F01_transcript/29961 | ACCGGACGTAAGATCATCATCG | ACTACACTCTTAGCGGCTTGTC |
| F01_transcript/43371 | CCAACACGATGAAACTGTGACC | GATGACTGGCTTGATGACATGC |
| F01_transcript/43680 | GGGCTTACATTGTTAGGCAAGC | CAAACACAGACAATGGCTCAGG |
| F01_transcript/27996 | TTGTCATTGGAGGACCTCATGG | CAATGTAAGCACCGCTTCTGTC |
| F01_transcript/42210 | TCCTCTGCTTCTACTTCAACGG | TACCAATCTGGCTTTGGAGGAC |
| F01_transcript/38601 | AGCTGGACTTGAATCTCTCTGC | GTGGCTTCAATCGAGTAAGCAC |
| F01_transcript/20597 | AGGAAGTTGCTGTGTCTAGTGG | ATGTCCTCATCCACACCCAAAG |
| F01_transcript/24731 | GGATGCTTGTCAAAGTGTTCCC | ACGGAGGCATAGCATGTAGAAG |
| F01_transcript/3597 | CCAACTGTTGGTGATGCAAGAG | GCGACGCTCTTGATTCTTGATC |
| F01_transcript/50209 | ACCTCGAATGTCTCCACCATTC | TCGAGGCTGCTCTTAGTTTACG |
| F01_transcript/47790 | GCTCTTCGCAAGGTTTATGACC | AATCCTGTCACATCCTCGAACC |
| F01_transcript/43592 | ACCGAGACACATGAGGAACTTC | ATTCAGCGGTAAAGGCTCTCTG |
| F01_transcript/47920 | ACCTGCAATGGAGGGACTTAAG | ATCGTGTAAGACATCCCTGAGC |
| F01_transcript/28873 | TGTACCTCAAGCGGTATGTCTG | GACGTGGTTATGTTGCAAGTCC |

**Table S6** continueed

| F01_transcript/20416 | CTATCTGCTTATTGCGCTTCCG | GAGCCATTGAGATCTTGTTCGC |
| --- | --- | --- |
| F01_transcript/18779 | ACTGGCGAGATCTCTTCGATTC | TTCTTCGACCATACACCCGATC |
| β-actin3 | TTTGGAGGCTCTGTGCTTTC | TGTACGGCATATGCTTGCTC |
| GAPB | AGCAGGGAAGCACATTCAAG | AGGAGCCAAACAGTTTGTGG |
| 18SRNA | TCGTATGGTTCTCGAGCTTCAG | AGCGCATCTGAAGCTTGTTG |
